# Esc2 and Rad57 contribute to RNA polymerase II distribution at highly transcribed genes

**DOI:** 10.26508/lsa.202603673

**Published:** 2026-10-12

**Authors:** Hiroshi Masumoto, Hideki Muto, Yohei Kurosaki, Hironori Niki

**Affiliations:** 1 Biomedical Research Support Center (BRSC), Nagasaki University School of Medicine, Nagasaki, Japan; 2 Research Center for Advanced Genomics, Nagasaki University School of Medicine, Nagasaki, Japan; 3 National Research Center for the Control and Prevention of Infectious Diseases, Nagasaki University , Nagasaki, Japan; 4 Microbial Physiology Laboratory, Department of Gene Function and Phenomics, National Institute of Genetics, Shizuoka, Japan

## Abstract

Transcription by RNA polymerase II (Pol II) must be tightly regulated to maintain gene expression homeostasis, yet how Pol II occupancy is shaped at highly active genes remains incompletely understood. Here, we examine the relationship between the homologous recombination–related factors Esc2 and Rad57 and Pol II distribution in budding yeast. Genome-wide analyses reveal that Esc2 and Rad57 preferentially associate with the gene bodies of highly transcribed housekeeping genes and are linked to a chromatin context that also includes the protein phosphatase Sit4 and its cofactor Rrd1. Loss of Esc2 and Rad57 is accompanied by increased Pol II occupancy within gene bodies, whereas the effect on steady-state RNA abundance is selective rather than uniform. Under the tested anchor-away conditions, acute Rad57 depletion in an *esc2*Δ background did not fully recapitulate the broader transcriptome imbalance and impaired growth observed after chronic Esc2/Rad57 loss. Together, our findings reveal an association between recombination-related factors and gene-body Pol II occupancy and suggest that Esc2/Rad57 loss is linked to transcription-associated chromatin changes in yeast.

## Abstract (precis)

Recombination-related factors are linked to gene-body Pol II occupancy and transcription-associated chromatin states at highly active genes in yeast.

## Introduction

Transcription by RNA polymerase II (Pol II) is a highly regulated process involving initiation, elongation, and termination (Kuehner et al, 2011; Jacobs & Schneider, 2024). In metazoans, promoter-proximal pausing is a well-characterized checkpoint in which Pol II transiently halts just downstream of transcription start sites, and this step is regulated by DSIF and NELF (Wada et al, 1998; Yamaguchi et al, 1999). This mechanism is central to the rapid and synchronous induction of genes such as those involved in stress responses and developmental programs. Recent comparative work further suggests that the evolutionary emergence of stable promoter-proximal pausing contributed to diversification of Pol II regulation across eukaryotes (Chivu et al, 2026).

In contrast, *Saccharomyces cerevisiae* lacks NELF and therefore does not establish stable promoter-proximal pausing as observed in higher eukaryotes (Gilchrist et al, 2008). Nevertheless, genome-wide analyses such as RNA sequencing (RNA-seq) and native elongating transcript sequencing consistently show that RNA synthesis is most abundant near transcription start sites, reflecting an elevated density of Pol II in regions proximal to transcription start sites (Gilchrist et al, 2008; Churchman & Weissman, 2011; Abril-Garrido et al, 2023). These observations suggest that budding yeast regulates the transition from initiation to productive elongation through mechanisms distinct from the pausing–release paradigm of metazoans. Core elongation factors, including Spt4-Spt5, the Paf1 complex, and chromatin regulators such as Spt6 and FACT, play central roles in this process (Rondón et al, 2004; Dronamraju et al, 2018). Thus, although promoter-proximal regulation is evident in yeast, its mechanistic basis differs fundamentally from that in metazoans.

Housekeeping genes encoding glycolytic enzymes and ribosomal proteins are among the most highly transcribed loci in yeast (Geisberg et al, 2024). Their expression is essential for energy metabolism and protein synthesis, and precise quantitative control of their transcriptional output is critical for cellular homeostasis. However, how cells prevent disproportionate accumulation of Pol II at these highly active genes—thereby potentially limiting balanced Pol II allocation across other transcription units—remains poorly understood.

Esc2 and Rad57 are homologous recombination (HR) factors traditionally recognized for their roles in maintaining genome stability. Esc2 contains a SUMO-like domain and functions in a distinct branch of the Rad51-mediated HR pathway, where it promotes Rad51 recruitment at perturbed replication forks, counteracts the anti-recombinase Srs2, and cooperates with the Mus81-Mms4 complex to resolve recombination intermediates (Mankouri et al, 2009; Parnas et al, 2010; Saugar et al, 2013). Rad57, together with its partner Rad55, forms a Rad51 paralog complex that stabilizes Rad51 nucleoprotein filaments on single-stranded DNA and promotes recombination under replication stress (Symington, 2002; Krogh & Symington, 2004). Although these factors are well established in DNA repair and replication-associated genome maintenance, our previous work showed that Esc2 and Rad57 are also required for GAGsi-mediated transcriptional silencing (Masumoto et al, 2024), suggesting that they may participate in transcription-associated chromatin regulation beyond canonical HR functions.

Recent studies have shown that transcriptional machinery, including Pol II, can become spatially concentrated at highly active regulatory elements or transcriptional hubs, leading to biased use of limited transcriptional resources within the nucleus (Cho et al, 2018; Sabari et al, 2018; Javed et al, 2022; Lyons et al, 2025). Such preferential concentration of Pol II has been proposed to stabilize gene expression programs, but it may also limit polymerase availability at other loci if left unbalanced. However, how Pol II occupancy is shaped not only at regulatory elements but also across the gene bodies of highly transcribed genes—particularly in organisms lacking canonical promoter-proximal pausing—remains poorly understood.

Here, we examine the relationship between the HR-related factors Esc2/Rad57 and Pol II occupancy at highly transcribed genes in budding yeast. Esc2 and Rad57 preferentially associate with gene bodies of highly transcribed housekeeping genes and overlap with a chromatin context that also includes the protein phosphatase Sit4 and its partner Rrd1. Combined loss of Esc2 and Rad57 is accompanied by increased Pol II occupancy within the gene bodies of a subset of highly expressed genes, whereas the effect on steady-state RNA output is selective rather than uniform. Acute depletion of Rad57 in an *esc2*Δ background causes only limited transcriptional changes, suggesting that the chronic *esc2*Δ *rad57*Δ phenotype reflects cumulative or indirect consequences of stable loss. Together, this work reveals an association between recombination-related factors, gene-body Pol II occupancy, and transcription-associated chromatin states in budding yeast.

## Results

### Esc2 and Rad57 preferentially associate with gene bodies of highly transcribed genes

**Figure 1.**
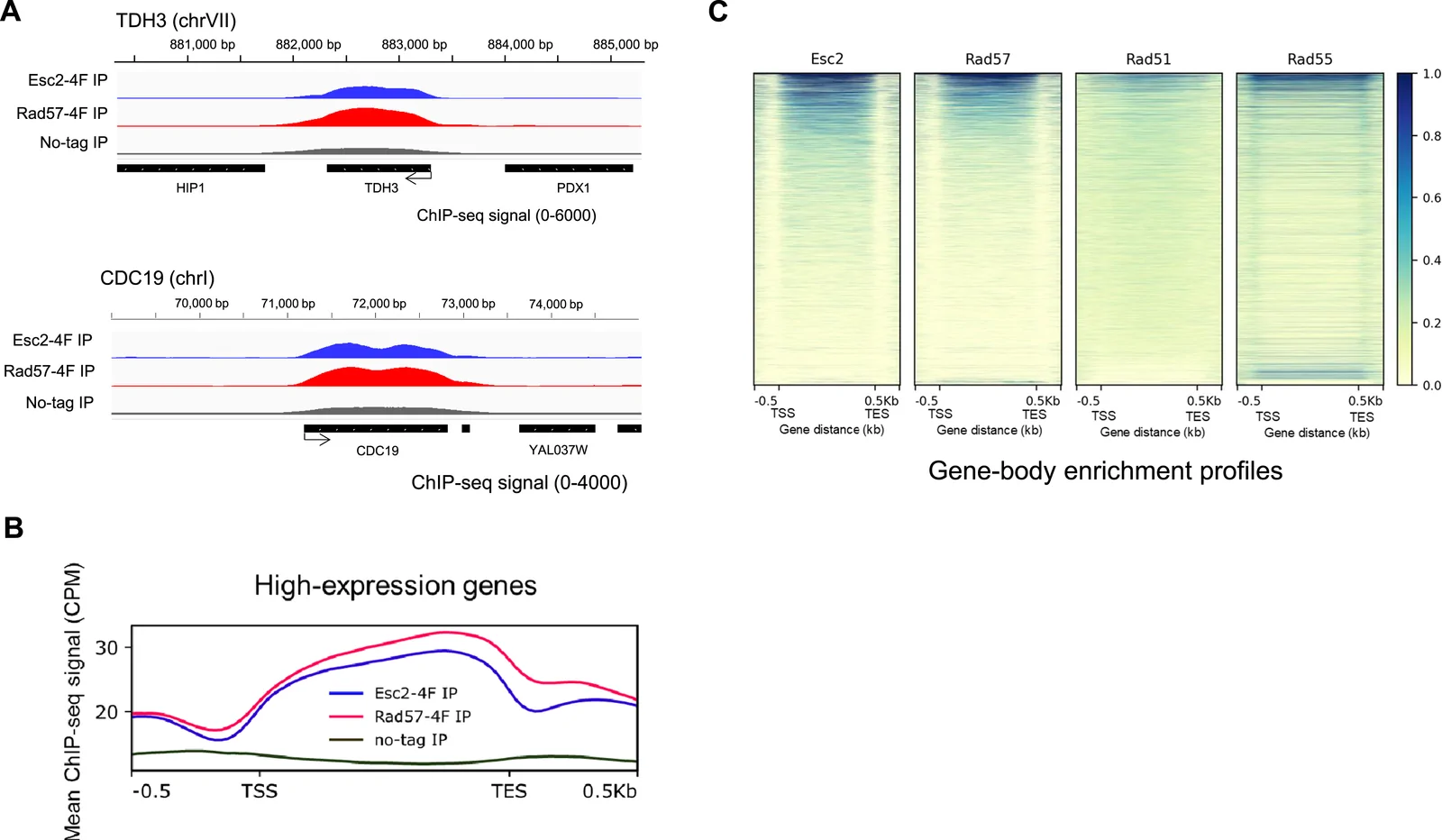
Esc2 and Rad57 preferentially associate with gene bodies of highly transcribed genes. **(A)** Representative genome-browser views showing Esc2-4Flag and Rad57-4Flag ChIP-seq signals together with no-tag IP controls at the highly transcribed glycolytic genes *TDH3* and *CDC19*. Although no-tag controls show the modest signal at these ChIP-prone loci, Esc2-4Flag and Rad57-4Flag display stronger enrichment across coding regions. **(B)** Metagene profiles of Esc2-4Flag, Rad57-4Flag, and no-tag IP control ChIP-seq signals over a cohort of highly expressed genes. Signals are plotted from 0.5 kb upstream of the TSS, across the gene body, to 0.5 kb downstream of the TES. **(C)** Heatmap comparison of gene-body enrichment profiles for Esc2, Rad57, Rad51, and Rad55 across analyzed genes. Esc2 and Rad57 show stronger gene body–biased enrichment patterns than the canonical HR factors Rad51 and Rad55. Heatmaps show ChIP-seq signals after subtraction of the common no-tag IP control. The corresponding unsubtracted no-tag IP signal is shown in Fig S1D. TSS, transcription start site; TES, transcription end site.

**Figure S1.**
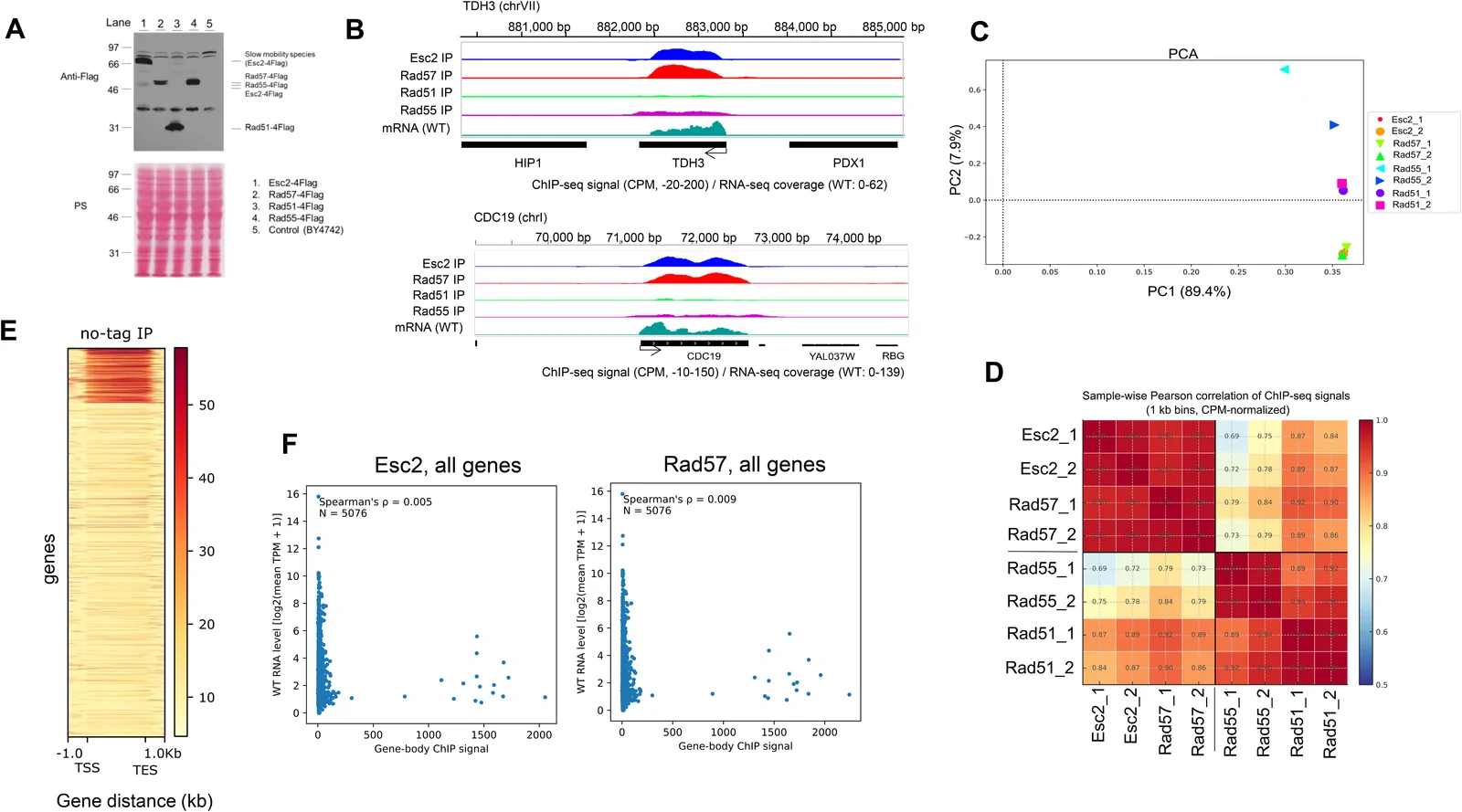
Genome-wide localization of Esc2 and Rad57. **(A)** Anti-Flag immunoblot showing detectability of Esc2-4Flag, Rad57-4Flag, Rad51-4Flag, and Rad55-4Flag in the strains used for ChIP-seq analyses. Ponceau S staining is shown as a loading control. **(B)** Representative genome-browser views at the highly transcribed genes *TDH3* and *CDC19*, comparing ChIP-seq signals for Esc2-4Flag, Rad57-4Flag, Rad51-4Flag, and Rad55-4Flag together with RNA-seq coverage in WT cells. Esc2 and Rad57 show clear gene body–biased enrichment, whereas Rad51 and Rad55 do not show comparable gene-body enrichment patterns. **(C)** PCA of ChIP-seq samples for Esc2-4Flag, Rad57-4Flag, Rad51-4Flag, and Rad55-4Flag. Biological replicates of each factor cluster closely, indicating good reproducibility. **(D)** Sample-wise Pearson’s correlation heatmap of the ChIP-seq datasets shown in (C), calculated from 1-kb genomic bins using CPM-normalized signals. **(E)** Heatmap showing the unsubtracted no-tag IP ChIP-seq signal over the same gene set and gene order used for the Flag-tagged factor heatmaps in Figs 1C, 4A, and S5C. This no-tag IP dataset was used as the common background control for the ChIP-seq analyses of Flag-tagged Esc2, Rad57, Rad51, Rad55, Sit4, Rrd1, and Ptc2. The color scale was adjusted to visualize the unsubtracted no-tag IP background signal. **(F)** Gene-by-gene comparisons between Esc2 or Rad57 gene-body ChIP-seq signal and steady-state RNA abundance in WT cells. Scatter plots show weak genome-wide correlations, despite preferential enrichment of Esc2 and Rad57 over highly expressed gene classes.

To examine how Esc2 and Rad57 associate with actively transcribed regions, we performed chromatin immunoprecipitation (ChIP) sequencing (ChIP-seq) and visualized representative loci using genome-browser views. At the highly transcribed glycolytic genes *TDH3* and *CDC19*, Esc2-4Flag and Rad57-4Flag showed enrichment across coding regions above the modest background observed in no-tag controls, whereas neighboring lowly expressed genes showed little or no signal (Fig 1A). Anti-Flag immunoblotting confirmed that Esc2-4Flag, Rad57-4Flag, Rad51-4Flag, and Rad55-4Flag were all detectable in the strains used for ChIP analyses, although Esc2-4Flag and Rad51-4Flag showed migration patterns that differed from simple size expectations (Fig S1A). At the same representative loci, Rad51 and Rad55—core HR factors and known partners of Rad57—did not show comparable gene-body enrichment patterns (Fig S1B). Genome-wide analyses further showed that Esc2 and Rad57 were enriched over highly expressed genes, as supported by metagene analysis (Fig 1B), whereas Rad51 and Rad55 showed distinct and weaker gene-body patterns in heatmap analyses (Fig 1C). PCA separated the Esc2-4Flag/Rad57-4Flag datasets from the Rad51-4Flag/Rad55-4Flag datasets, with biological replicates clustering closely for each factor (Fig S1C). Pairwise correlation analysis further confirmed the reproducibility of biological replicates and supported the distinction between Esc2/Rad57 and Rad51/Rad55 ChIP-seq profiles (Fig S1D). To account for background ChIP-seq signals at a subset of loci, the Flag-tagged factor heatmaps were generated after subtraction of the common no-tag IP control; the corresponding unsubtracted no-tag IP signal is shown in Fig S1E. Together, these results indicate that Esc2 and Rad57 display a gene body–biased chromatin association that is not shared by the canonical HR factors Rad51 and Rad55.

Direct gene-by-gene comparison further showed that Esc2 and Rad57 occupancy correlated only weakly with steady-state RNA abundance across all genes (Fig S1F). Nevertheless, metagene analysis over a high-expression gene cohort revealed enrichment of both factors across gene bodies (Fig 1B). These observations indicate that Esc2/Rad57 preferentially associate with highly active transcription units, whereas their occupancy does not follow a simple monotonic relationship with RNA output.

### Esc2/Rad57-associated gene classes show selective RNA changes in *esc2*Δ *rad57*Δ cells

**Figure 2.**
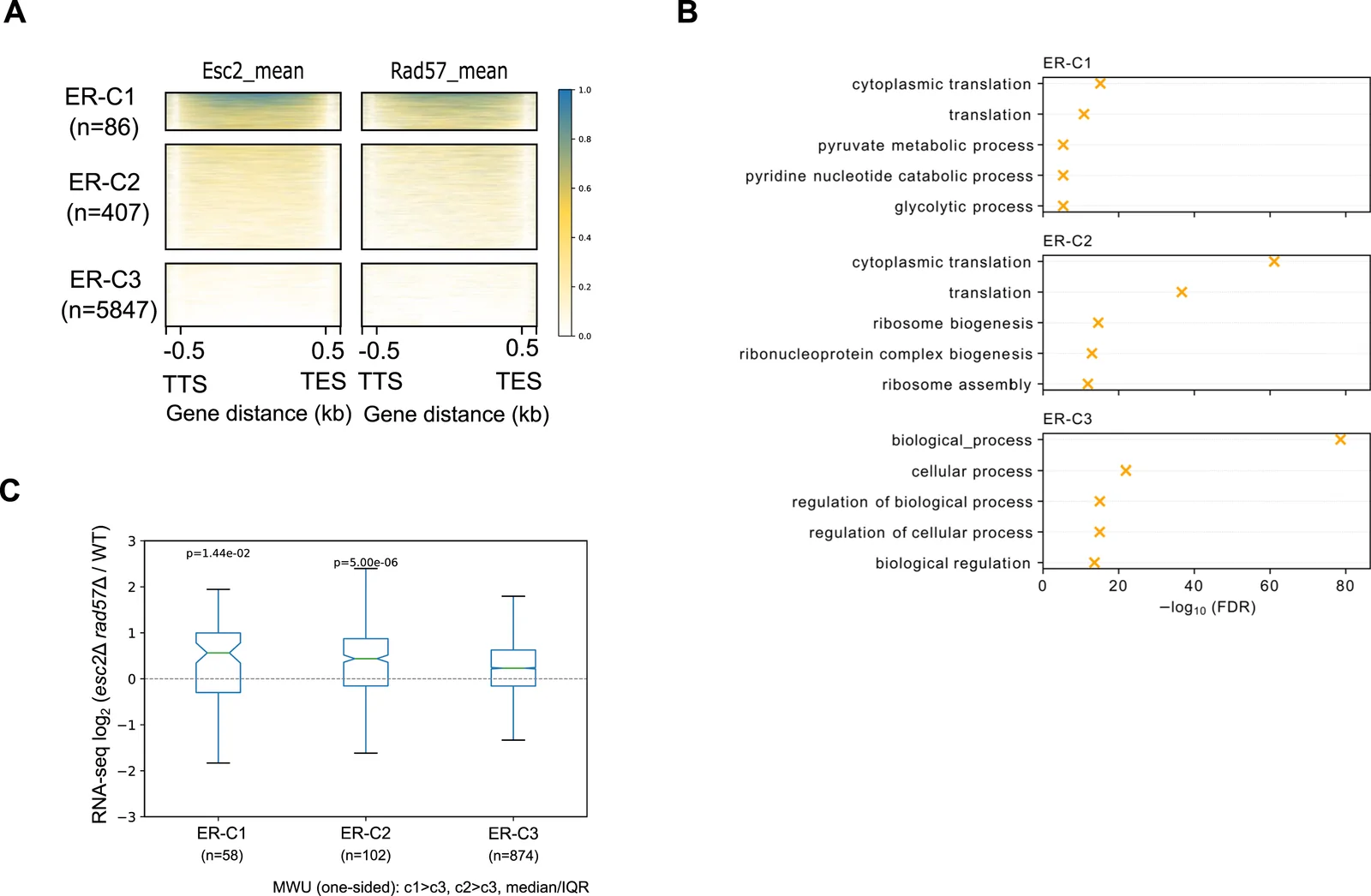
Esc2/Rad57-defined gene classes show selective RNA changes in *esc2*Δ *rad57*Δ cells. **(A)** Genes were classified into three Esc2/Rad57-defined clusters (ER-C1 to ER-C3) according to the Esc2/Rad57 ChIP-seq signal intensity across gene bodies. Heatmaps show the mean Esc2 and Rad57 ChIP-seq signals for each cluster. Numbers indicate the total genes assigned to each cluster. **(B)** GO enrichment analysis of each Esc2/Rad57-defined cluster. ER-C1 and ER-C2 are enriched for highly expressed housekeeping functions, including cytoplasmic translation, ribosome biogenesis, and central carbon metabolism. **(C)** Box plots showing RNA-seq changes in *esc2*Δ *rad57*Δ cells relative to WT across ER-C1, ER-C2, and ER-C3 genes. ER-C1 and ER-C2 genes show significantly greater steady-state RNA changes than ER-C3 genes. Numbers indicate genes retained for RNA-seq comparison.

To characterize the genomic distribution of Esc2- and Rad57-bound genes, we classified analyzed genes into three Esc2/Rad57-defined clusters (ER-C1 to ER-C3) according to Esc2/Rad57 ChIP-seq signal intensity (Fig 2A). Functional enrichment analysis revealed that ER-C1 genes, which displayed the strongest binding, were enriched for pathways that are typically among the most highly expressed in exponentially growing yeast, including cytoplasmic translation and central carbon metabolism. ER-C2 genes were also enriched for highly expressed housekeeping functions, particularly translation- and ribosome biogenesis–related pathways, whereas ER-C3 genes showed weaker binding and less coherent functional enrichment (Fig 2B). These data further support the preferential association of Esc2 and Rad57 with highly transcribed gene classes.

**Figure S2.**
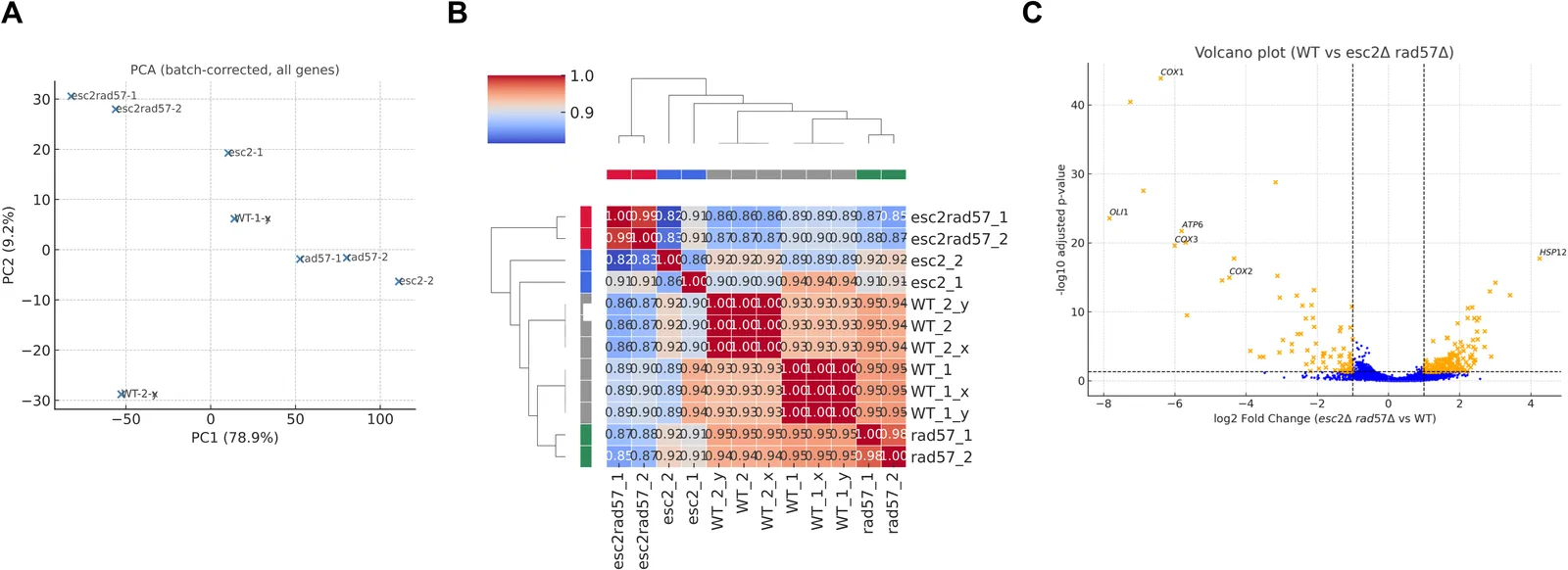
Quality control and transcriptome analysis of RNA-seq datasets. **(A)** PCA of RNA-seq samples from WT, *esc2*Δ, *rad57*Δ, and *esc2*Δ *rad57*Δ cells after batch correction, based on all genes. The *esc2*Δ *rad57*Δ samples are separated from WT and the single mutants. **(B)** Sample-wise Pearson’s correlation heatmap of the RNA-seq datasets shown in (C), with hierarchical clustering. **(C)** Volcano plot showing differential gene expression in *esc2*Δ *rad57*Δ versus WT. Up-regulated and down-regulated genes are highlighted according to the significance and fold-change thresholds used in the analysis.

To assess RNA changes associated with Esc2/Rad57 loss, we compared RNA-seq profiles of *esc2*Δ, *rad57*Δ, and *esc2*Δ *rad57*Δ cells with wild-type (WT) cells. PCA separated the *esc2*Δ *rad57*Δ transcriptome from WT and from the single mutants (Fig S2A). Pairwise correlation analysis further showed high similarity among WT and *esc2*Δ *rad57*Δ biological replicates, whereas the *esc2*Δ and *rad57*Δ single-mutant datasets showed more limited transcriptome differences from WT and greater inter-replicate variability (Fig S2B). Together, these analyses indicated that the double mutant showed a more pronounced transcriptome shift than the single mutants (Fig S2A–C).

Functional enrichment analysis of genes up-regulated in *esc2*Δ *rad57*Δ further showed overrepresentation of highly expressed housekeeping pathways, including translation/ribosome biogenesis and glycolytic metabolism (Fig S6A).

We next examined whether altered RNA levels preferentially occurred in genes associated with Esc2 and Rad57. Genes in ER-C1 and ER-C2 showed significantly greater RNA-seq changes than ER-C3 genes (Fig 2C), indicating that Esc2/Rad57 occupancy is associated with highly active gene-body chromatin contexts in which steady-state RNA changes become apparent only for a subset of genes. Because RNA-seq reflects the combined contributions of RNA synthesis and RNA decay, these data do not by themselves distinguish transcriptional from posttranscriptional effects. Rather, they indicate that *esc2*Δ *rad57*Δ cells show selective changes in the steady-state expression of gene sets associated with strong Esc2/Rad57 chromatin occupancy, thereby providing a transcriptome-level context for subsequent analyses of Pol II occupancy.

### *esc2*Δ *rad57*Δ cells show increased Pol II gene-body occupancy at highly transcribed loci

**Figure 3.**
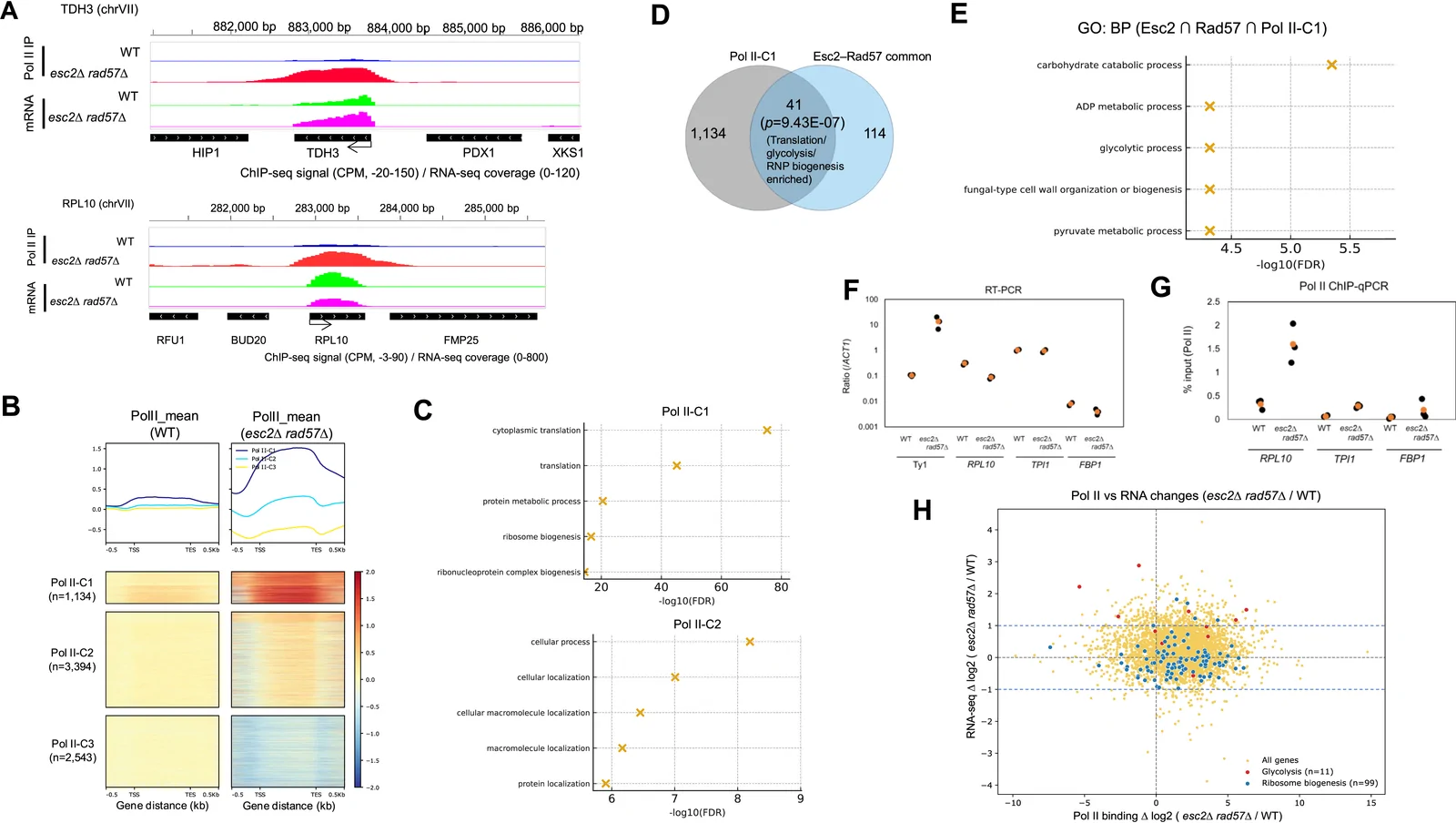
*esc2*Δ *rad57*Δ cells show increased Pol II gene-body occupancy at highly transcribed loci. **(A)** Representative genome-browser views of Pol II ChIP-seq and RNA-seq signals at the highly transcribed *TDH3* and *RPL10* loci in WT and *esc2*Δ *rad57*Δ cells. Pol II ChIP-seq tracks show mean CPM-normalized signals after subtraction of the no-antibody IP control. The double mutant shows increased Pol II signal across the coding region. Each locus was displayed using the indicated scale. **(B)** Metagene profiles and heatmaps of Pol II ChIP-seq signals across genes grouped into three Pol II–defined clusters (Pol II–C1 to Pol II–C3) based on the magnitude and spatial distribution of Pol II accumulation in *esc2*Δ *rad57*Δ relative to WT. Upper panels show mean Pol II profiles in WT and *esc2*Δ *rad57*Δ, and lower panels show the corresponding heatmaps for each cluster. Numbers indicate the total genes assigned to each cluster. **(C)** GO enrichment analysis of Pol II–C1 and Pol II–C2 genes. Pol II–C1 is enriched for highly expressed housekeeping functions, including cytoplasmic translation, ribosome biogenesis, and protein metabolic processes. **(D)** Overlap between Pol II–C1 genes and Esc2/Rad57 common genes. The overlap is greater than expected by chance and includes genes enriched for glycolysis- and ribosome biogenesis–related functions. **(E)** GO enrichment analysis of the overlap gene set shown in panel D. **(F)** RT–qPCR analysis of representative transcripts (Ty1, *RPL10*, *TPI1*, and *FBP1*) in WT and *esc2*Δ *rad57*Δ cells. Transcript levels were normalized to *ACT1* and plotted on a log scale. Individual biological replicates are shown as dots, and orange circles indicate the mean of three biological replicates. *FBP1* was included as a low-expression control gene that did not show increased RNA abundance in *esc2*Δ *rad57*Δ cells. Ty1 primers were designed against a consensus sequence shared among 31 Ty1 elements. **(G)** Pol II ChIP-qPCR analysis at *RPL10*, *TPI1*, and *FBP1* in WT and *esc2*Δ *rad57*Δ cells. Signals are shown as % input. Individual biological replicates are shown as dots, and orange circles indicate the mean of three biological replicates. *FBP1* was included as a control locus that did not show increased Pol II occupancy or RNA abundance in *esc2*Δ *rad57*Δ cells in the genome-wide datasets. **(H)** Scatter plot comparing changes in Pol II gene-body occupancy and steady-state RNA abundance in *esc2*Δ *rad57*Δ relative to WT. Large increases in Pol II occupancy are often associated with only modest RNA changes, indicating that elevated Pol II gene-body occupancy does not uniformly translate into increased steady-state RNA output.

**Figure S3.**
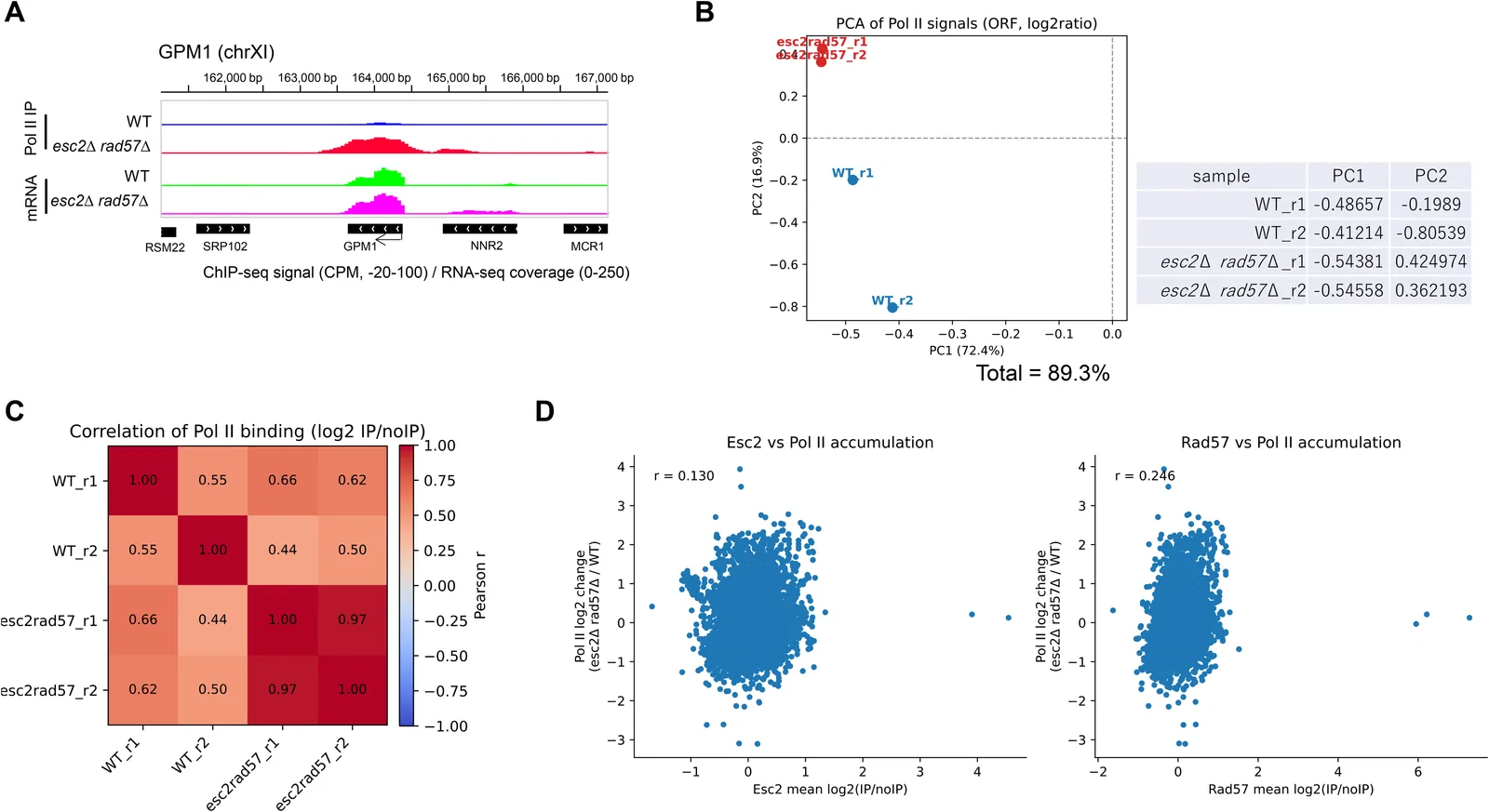
Supplementary analyses of Pol II ChIP-seq signals. **(A)** Representative genome-browser views of Pol II ChIP-seq and RNA-seq signals at the *GPM1* locus in WT and *esc2*Δ *rad57*Δ cells. This locus shows increased Pol II signal across the coding region in the double mutant, similar to the representative locus shown in Fig 3A. **(B)** PCA of Pol II ChIP-seq signals calculated over ORF regions using log2 IP/noIP ratios. **(C)** Sample-wise Pearson’s correlation heatmap of Pol II ChIP-seq signals shown in (B). The *esc2*Δ *rad57*Δ biological replicates showed high similarity, whereas the WT replicates showed greater variability. **(D)** Gene-by-gene comparison between Esc2 or Rad57 mean gene-body ChIP-seq signal and Pol II accumulation in *esc2*Δ *rad57*Δ cells relative to WT. Both comparisons show only weak positive correlation, indicating that the relationship between Esc2/Rad57 occupancy and Pol II accumulation is not captured by a simple monotonic proportional model.

To examine Pol II occupancy in the absence of Esc2 and Rad57, we performed Pol II ChIP-seq in WT and *esc2*Δ *rad57*Δ cells. Genome-browser views at representative highly transcribed loci, including *TDH3* and *RPL10*, revealed increased Pol II signal across coding regions in the double mutant compared with WT (Figs 3A and S3A). The *RPL10* locus was also examined by Pol II ChIP-qPCR in the locus-specific analysis described below (Fig 3G). Principal component and pairwise correlation analyses of Pol II ChIP-seq signals showed close clustering of the *esc*2Δ *rad57*Δ biological replicates, whereas the WT replicates showed greater variability (Fig S3B and C). The gene-level summary of Pol II accumulation, Pol II–defined cluster assignments, and associated enrichment analyses are provided in Table S1. We therefore interpreted these data cautiously and focused on reproducible gene body–biased occupancy patterns rather than absolute signal differences between individual samples.

Table S1. Gene-level summary and enrichment analyses related to Pol II accumulation in *esc2*Δ *rad57*Δ cells.

Metagene analysis further showed gene body–biased Pol II signal patterns in *esc2*Δ *rad57*Δ cells compared with WT, whereas promoter-proximal regions displayed comparatively modest differences (Fig 3B). Because these ChIP-seq datasets were not spike-in–normalized, we interpreted these profiles primarily in terms of relative Pol II distribution patterns rather than absolute differences in Pol II occupancy between strains or clusters. Based on the magnitude and spatial distribution of Pol II signal changes, we classified genes into three Pol II–defined clusters (Pol II–C1 to Pol II–C3). Pol II–C1, which showed the strongest gene body–biased Pol II signal increase, was enriched for highly expressed housekeeping functions, including cytoplasmic translation, ribosome biogenesis, and protein metabolic processes. Pol II–C2 showed a weaker but still detectable gene body–biased increase, whereas Pol II–C3 did not show a comparable gene body–biased increase (Fig 3B and C).

We next compared genes exhibiting increased Pol II occupancy with genes associated with Esc2 and Rad57. Although the binary overlap between Pol II–C1 and Esc2/Rad57 common genes was limited, it was greater than expected by chance, and the overlapping genes were enriched for glycolysis- and ribosome biogenesis–related functions (Fig 3D and E). Thus, Esc2/Rad57-associated loci are preferentially represented among genes showing increased Pol II gene-body occupancy in *esc2*Δ *rad57*Δ cells.

Locus-specific analyses provided additional support for this interpretation. At representative genes, Pol II ChIP-qPCR showed a clear increase at *RPL10*, whereas *TPI1* showed little or no increase, and the corresponding RNA changes measured by RT-qPCR were gene-dependent rather than uniform (Fig 3F and G). *FBP1* was included as a control locus because genome-wide Pol II ChIP-seq and RNA-seq datasets showed no increase in Pol II occupancy or RNA abundance at this locus in *esc2*Δ *rad57*Δ cells. Genome-wide comparison likewise showed that large changes in Pol II occupancy were often associated with only modest changes in RNA abundance (Fig 3H). Direct gene-by-gene comparison further revealed only a weak positive correlation between Esc2/Rad57 occupancy and Pol II occupancy changes in *esc2*Δ *rad57*Δ cells (Fig S3D), indicating that this relationship is not captured by a simple globally proportional model. These results indicate that elevated Pol II occupancy within gene bodies does not uniformly translate into increased steady-state RNA output.

**Figure S4.**
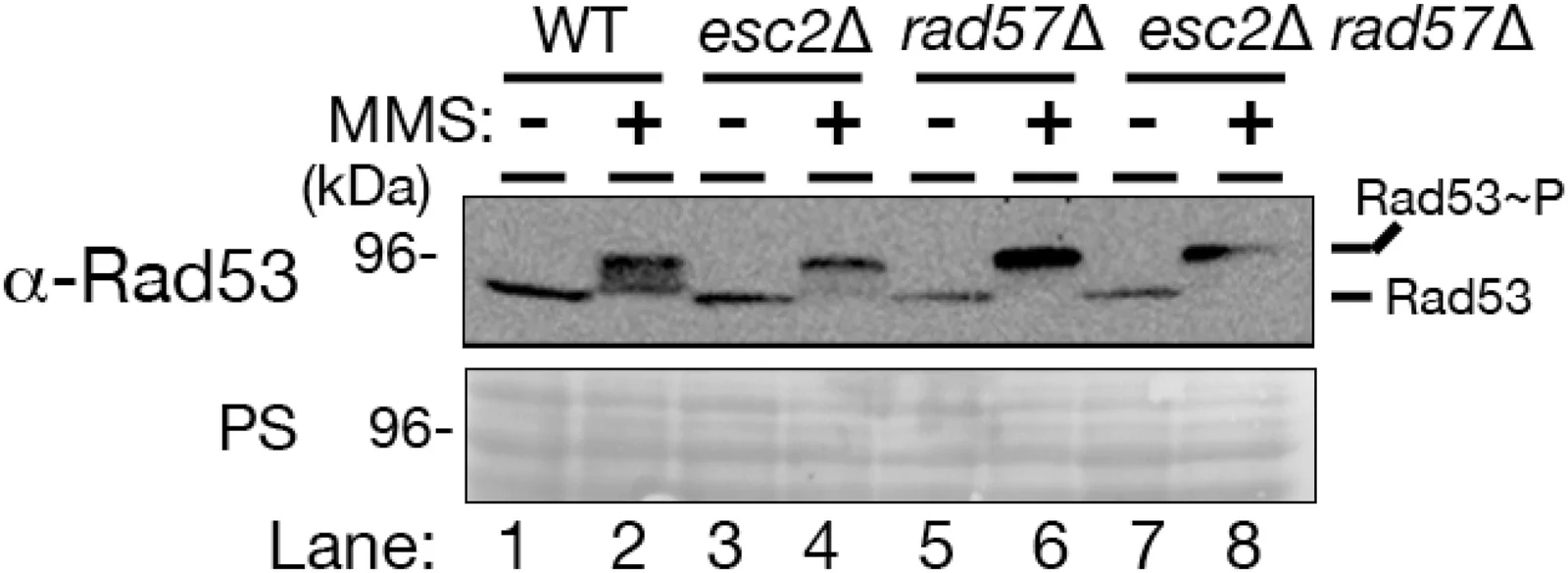
*esc2*Δ *rad57*Δ cells do not show detectable Rad53 activation under these conditions. Western blot analysis of Rad53 phosphorylation in WT, *esc2*Δ, *rad57*Δ, and *esc2*Δ *rad57*Δ cells. Cells were cultured in YPD at 25°C to a mid-log phase and split equally. One half was treated with 0.033% MMS for 3 h, and the other half was left untreated (−). Proteins were extracted by the post-alkaline method (Kushnirov, 2000) and analyzed by 10% SDS–PAGE. Rad53 was detected using an anti-Rad53 antibody (a gift from Dr. Hohn FX Diffley, The Crick Institute, UK). A mobility shift corresponding to Rad53 phosphorylation was observed after MMS treatment in all strains, but not under untreated conditions, indicating that *esc2*Δ *rad57*Δ cells do not show overt checkpoint activation during normal growth under the conditions tested.

Because increased Pol II occupancy could, in principle, also arise indirectly from DNA damage signaling, we examined activation of the checkpoint kinase Rad53. Under normal growth conditions, *esc2*Δ *rad57*Δ cells did not show an obvious Rad53 mobility shift, indicating that the Pol II occupancy changes described here are not accompanied by overt checkpoint activation under these conditions (Fig S4).

### Sit4 and Rrd1 are associated with Esc2/Rad57-enriched gene bodies and Pol II occupancy patterns

**Figure S5.**
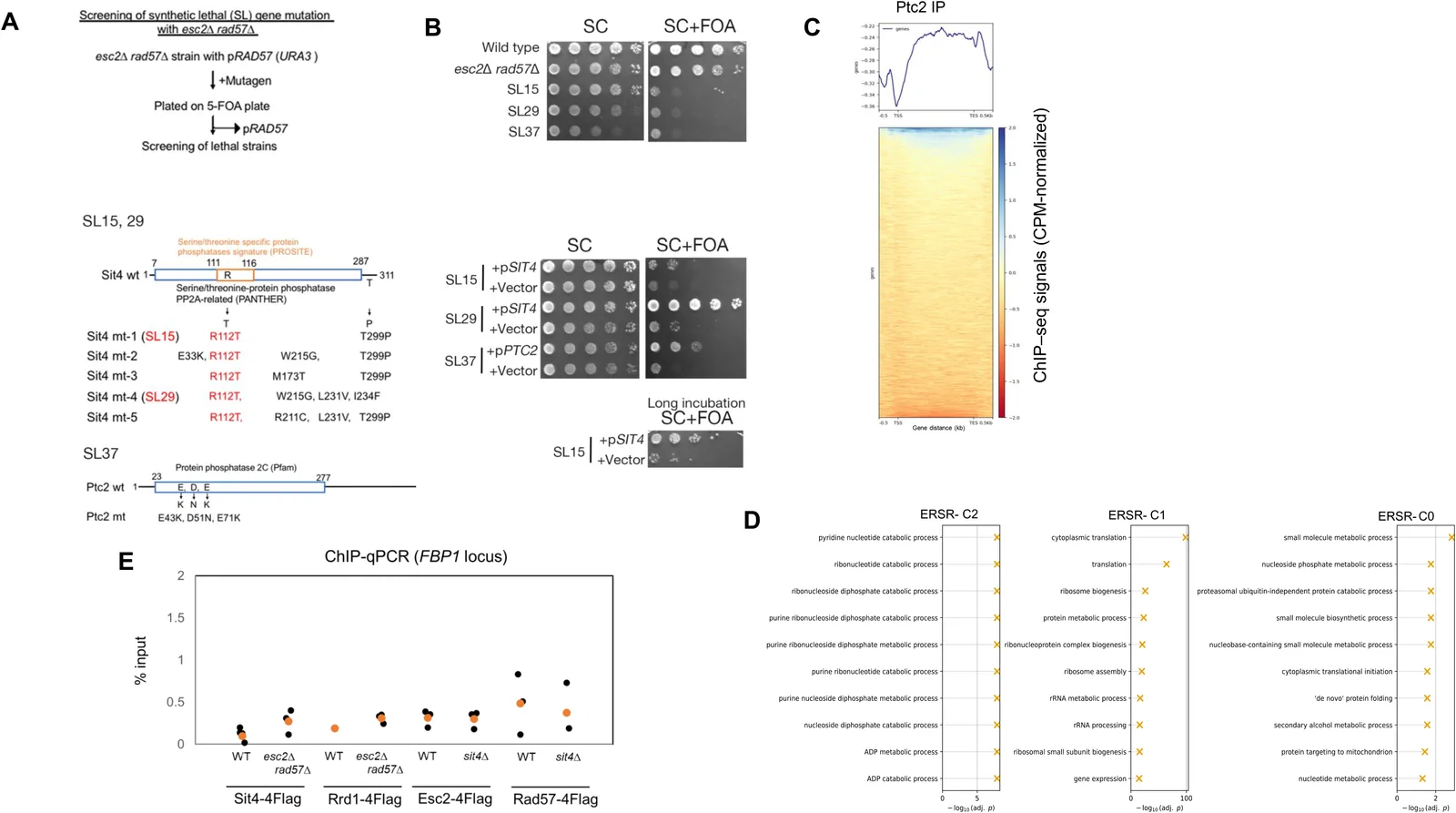
Supporting data related to Sit4/Ptc2 genetic screening and Ptc2 localization. **(A)** Synthetic-lethal screening of *esc2*Δ *rad57*Δ identifies point mutations in *SIT4* and *PTC2*. Schematic overview of the screening strategy and the positions of point mutations recovered in *SIT4* and *PTC2*. This screen prompted further analysis of Sit4 as a candidate factor functionally linked to Esc2/Rad57. **(B)** Genetic complementation of synthetic-lethal mutations. Colony-growth assays showing that plasmid-borne *SIT4* or *PTC2* complements the corresponding mutations isolated from the synthetic-lethal screen in *esc2*Δ *rad57*Δ cells. **(C)** Genome-wide localization of Ptc2. Mean profile and heatmap of Ptc2 ChIP-seq signals aligned relative to gene architecture. Ptc2 does not show a comparably clear gene-body enrichment pattern across Pol II–transcribed genes and was therefore not pursued further in the main gene body–focused analyses. Heatmaps show ChIP-seq signals after subtraction of the common no-tag IP control. The corresponding unsubtracted no-tag IP signal is shown in Fig S1D. **(D)** GO enrichment analysis of genes in the ERSR-C2, ERSR-C1, and ERSR-C0 clusters defined by Esc2/Rad57/Sit4/Rrd1 ChIP-seq signal patterns. ERSR-C2 and ERSR-C1 genes were enriched for highly expressed housekeeping functions, including cytoplasmic translation, ribosome biogenesis, and related biosynthetic pathways. The top enriched biological process terms are shown for each cluster. **(E)** ChIP-qPCR analysis of the indicated Flag-tagged proteins at *FBP1*, used as a low-signal control locus. Sit4-4Flag and Rrd1-4Flag were analyzed in WT and *esc2*Δ *rad57*Δ cells, and Esc2-4Flag and Rad57-4Flag were analyzed in WT and *sit4*Δ cells. Signals are shown as % input. Individual biological replicates are shown as dots, and orange circles indicate the mean. n = 3 except for Rrd1-4Flag WT, for which one biological sample was available. No statistical test was performed.

**Figure 4.**
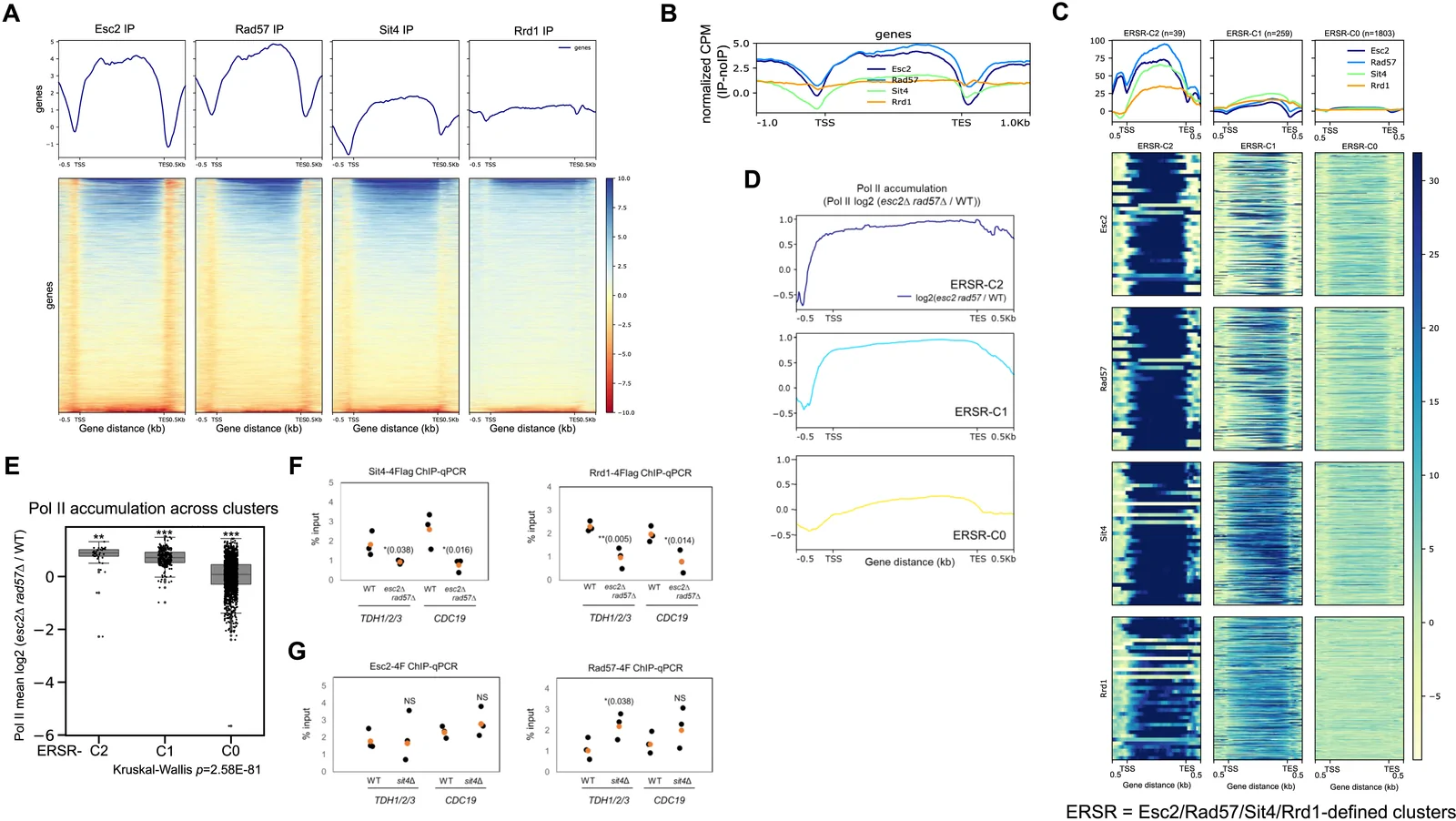
Sit4 and Rrd1 are associated with Esc2/Rad57-enriched gene bodies, and this association is reduced in *esc2*Δ *rad57*Δ. **(A)** Genome-wide ChIP-seq profiles and heatmaps for Esc2, Rad57, Sit4, and Rrd1 across analyzed genes aligned relative to gene architecture. Signals are shown from 0.5 kb upstream of the TSS to 0.5 kb downstream of the TES and are displayed as IP-noIP, CPM-normalized values. Heatmaps show ChIP-seq signals after subtraction of the common no-tag IP control. The corresponding unsubtracted no-tag IP signal is shown in Fig S1D. **(B)** Mean metagene profiles of Esc2, Rad57, Sit4, and Rrd1 across analyzed genes, showing gene body–biased enrichment for all four factors. **(C)** Genes were grouped into three Esc2/Rad57/Sit4/Rrd1-defined clusters (ERSR-C2, ERSR-C1, and ERSR-C0) based on ChIP-seq signal intensity and distribution across gene bodies. Upper panels show the mean profiles for each cluster, and lower panels show the corresponding heatmaps. ERSR clusters denote Esc2/Rad57/Sit4/Rrd1-defined clusters. **(D)** Metagene profiles of Pol II accumulation in *esc2*Δ *rad57*Δ relative to WT across ERSR-defined clusters, shown as log2(*esc2*Δ *rad57*Δ/WT). Clusters with stronger four-factor association show larger increases in gene-body Pol II occupancy. **(E)** Box plots summarizing Pol II gene-body occupancy changes across ERSR-defined clusters. Values are shown as mean log2(*esc2*Δ *rad57*Δ/WT) for genes in each cluster. Statistical significance was evaluated by the Kruskal–Wallis test; the *P*-value is indicated in the panel. **(F, G)** ChIP-qPCR analysis of Sit4-4Flag and Rrd1-4Flag in WT and *esc2*Δ *rad57*Δ cells (F), and Esc2-4Flag and Rad57-4Flag in WT and *sit4*Δ cells (G), at representative highly transcribed gene loci (*TDH1/2/3* and *CDC19*). Signals are shown as % input. Individual biological replicates are shown as dots, and orange circles indicate the mean (n = 3). The *TDH1/2/3* primer set detects conserved sequences shared among the *TDH* paralogs and was used to assess the *TDH* paralogous loci collectively. *P*-values were calculated using an unpaired one-tailed *t* test. *P* < 0.05 (*), *P* < 0.01 (**), and *P* < 0.001 (***). TSS, transcription start site; TES, transcription end site.

To identify factors genetically or functionally connected to Esc2 and Rad57, we performed a synthetic-lethal screen in the *esc2*Δ *rad57*Δ background. Among the factors reproducibly isolated in this screen was the protein phosphatase Sit4 (Fig S5A and B). Because Sit4 has been implicated in transcription-associated regulation and functions together with the prolyl isomerase Rrd1 (Douville et al, 2004; Alfatah et al, 2021; Kashif et al, 2023), we asked whether these factors are associated with Esc2/Rad57-enriched gene bodies. Genome-wide ChIP-seq analyses showed that like Esc2 and Rad57, Sit4 and Rrd1 were preferentially associated with gene bodies of highly transcribed genes, although the Rrd1 pattern was weaker and less sharply gene body–centered than that of Esc2, Rad57, and Sit4 (Fig 4A and B). In contrast, Ptc2, another phosphatase recovered in the screen, did not show a comparably clear gene-body enrichment pattern and was not pursued further in the main analyses (Fig S5C).

To characterize the extent and patterns of co-association among these factors, we grouped genes into three Esc2/Rad57/Sit4/Rrd1-defined clusters (ERSR-C2, ERSR-C1, and ERSR-C0) based on their ChIP-seq signal intensity and distribution across gene bodies (Fig 4C). Gene Ontology (GO) enrichment analyses of the Esc2/Rad57/Sit4/Rrd1-defined gene clusters are provided in Table S2. These clusters differed primarily in the strength of four-factor association across gene bodies rather than in promoter-proximal enrichment. Consistent with this, genes in ERSR-C2 and ERSR-C1 were enriched for highly expressed housekeeping functions, including cytoplasmic translation, ribosome biogenesis, and related biosynthetic pathways (Fig S5D).

Table S2. GO enrichment analyses of Esc2/Rad57/Sit4/Rrd1-defined gene clusters (related to Fig 4).

We next examined how these four-factor association patterns relate to Pol II occupancy in *esc2*Δ *rad57*Δ cells. Metagene analysis showed that gene classes with stronger Esc2/Rad57/Sit4/Rrd1 association exhibited larger increases in Pol II gene-body occupancy in the double mutant (Fig 4D). This relationship was further supported by quantitative comparison across clusters, which showed significantly greater Pol II occupancy changes in ERSR-C2 and ERSR-C1 than in ERSR-C0 (Fig 4E). Thus, genes most strongly associated with Esc2, Rad57, Sit4, and Rrd1 are preferentially represented among loci showing increased Pol II occupancy in *esc2*Δ *rad57*Δ cells.

To examine whether Sit4 and Rrd1 association with these loci is altered in *esc2*Δ *rad57*Δ cells, we performed ChIP-qPCR at representative highly transcribed genes (*TDH1/2/3* and *CDC19*). ChIP-qPCR signals for Sit4-4Flag and Rrd1-4Flag were reduced in *esc2*Δ *rad57*Δ cells compared with WT (Fig 4F), indicating that Sit4 and Rrd1 association with these representative loci is altered in the absence of Esc2 and Rad57. In contrast, ChIP-qPCR analysis showed that Esc2-4Flag and Rad57-4Flag association at the same loci was not markedly affected by *sit4*Δ (Fig 4G). *FBP1* was also analyzed as a low-signal control locus using the available ChIP DNA samples and did not show a background-specific increase in the ChIP-qPCR signal (Fig S5E). These results indicate that Sit4/Rrd1 association at these representative loci is reduced in *esc2*Δ *rad57*Δ cells, whereas Esc2/Rad57 association is not markedly affected by *sit4*Δ.

Together, these findings indicate that Sit4 and Rrd1 are associated with an Esc2/Rad57-enriched gene-body chromatin context at highly transcribed genes and that this four-factor association pattern is linked to increased Pol II occupancy observed in *esc2*Δ *rad57*Δ cells.

### Reduced mitochondrial transcripts in *esc2*Δ *rad57*Δ are associated with mitochondrial DNA loss

**Figure S6.**
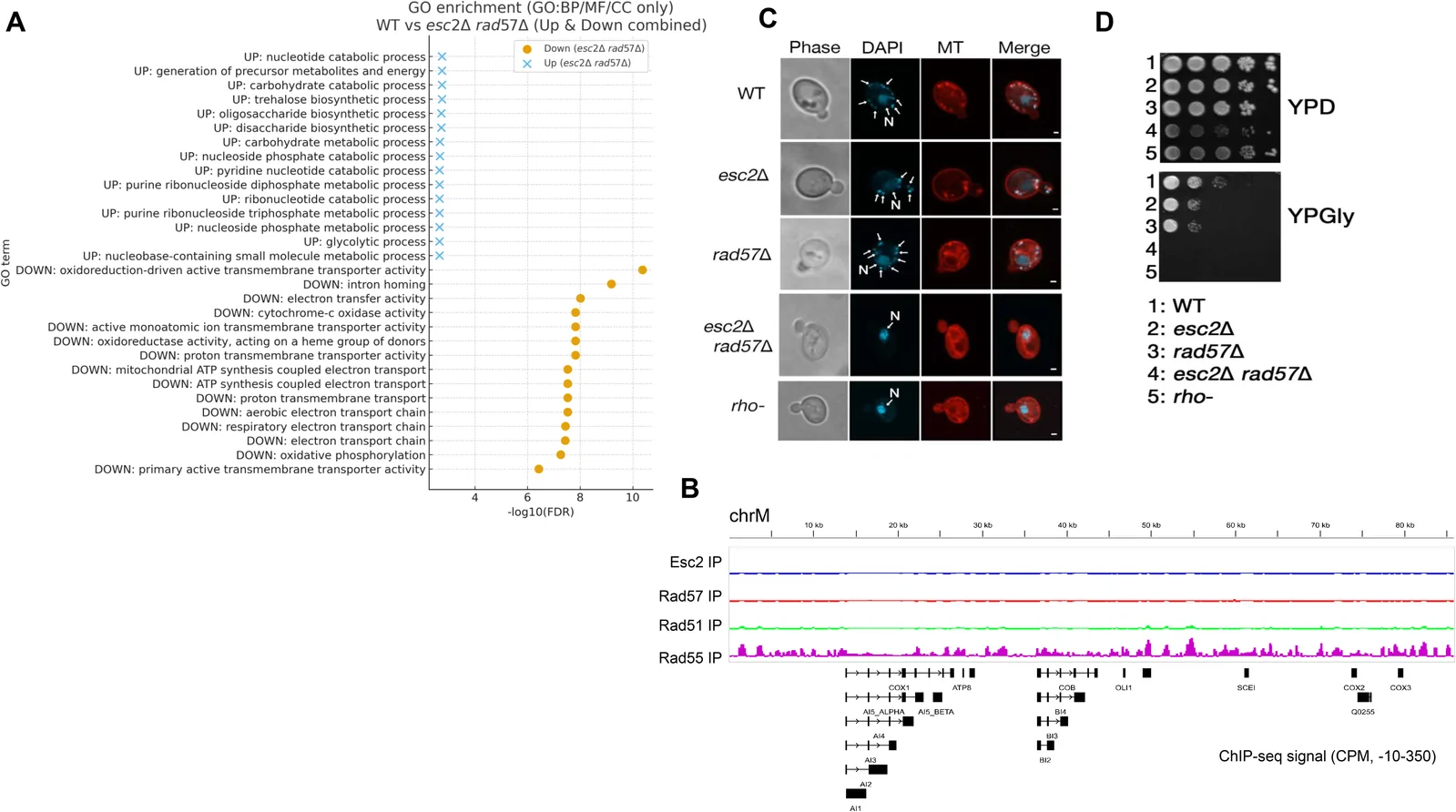
Mitochondrial defects and respiratory deficiency in *esc2*Δ *rad57*Δ cells. **(A)** GO enrichment analysis of genes differentially expressed in *esc2*Δ *rad57*Δ cells relative to WT. Down-regulated genes were strongly enriched for mitochondrial functions, including oxidative phosphorylation and electron transport–related pathways, whereas up-regulated genes were enriched for carbohydrate and nucleotide metabolic processes (gene lists in Table S2D). **(B)** Genome-browser view of Esc2-4Flag, Rad57-4Flag, Rad51-4Flag, and Rad55-4Flag ChIP-seq signals across mtDNA (chrM). Esc2 and Rad57 do not show detectable enrichment across mtDNA, consistent with the idea that the mitochondrial defects of *esc2*Δ *rad57*Δ cells do not reflect direct chromatin association at mitochondrial loci. **(C)** Detection of mtDNA by fluorescence microscopy in WT and mutant strains. Phase, phase-contrast image; DAPI, nuclear and mtDNA stained with DAPI; MT, mitochondria stained with MitoTracker Red CMXRos; Merge, merged image. Arrows indicate mtDNA foci, and N denotes nuclear DNA. The *esc2*Δ *rad57*Δ mutant lacks DAPI-positive mitochondrial nucleoids, resembling *rho*^−^ cells. Scale bars, 2 μm. **(D)** Growth phenotypes on fermentable (YPD) and nonfermentable (YPG) media. Cells were spotted on agar plates in serial dilutions. Growth on YPD indicates general viability, whereas failure to grow on YPG indicates respiratory deficiency consistent with a rho^−^-like phenotype.

RNA-seq analysis further revealed a prominent class of down-regulated genes in *esc2*Δ *rad57*Δ cells that was enriched for mitochondrial functions, particularly oxidative phosphorylation and electron transport–related pathways (Fig S6A). Because many of these transcripts are encoded by the mitochondrial genome, we asked whether this decrease might reflect mitochondrial DNA (mtDNA) instability rather than direct transcriptional regulation by Esc2/Rad57. Consistent with this interpretation, Esc2 and Rad57 did not show detectable enrichment on mtDNA (Fig S6B). Fluorescence microscopy further showed that *esc2*Δ *rad57*Δ cells lacked mitochondrial nucleoids, resembling *rho*^*-*^ cells (Fig S6C), and the double mutant failed to grow on glycerol medium (Fig S6D). Together, these results suggest that reduced mitochondrial transcripts in *esc2*Δ *rad57*Δ cells primarily reflect secondary consequences of mtDNA loss rather than direct regulation by Esc2/Rad57 (Table S3).

Table S3. Gene-level summary of Esc2/Rad57 chromatin association and RNA-seq changes.

### Chronic loss of Esc2/Rad57 is associated with slow growth

**Figure 5.**
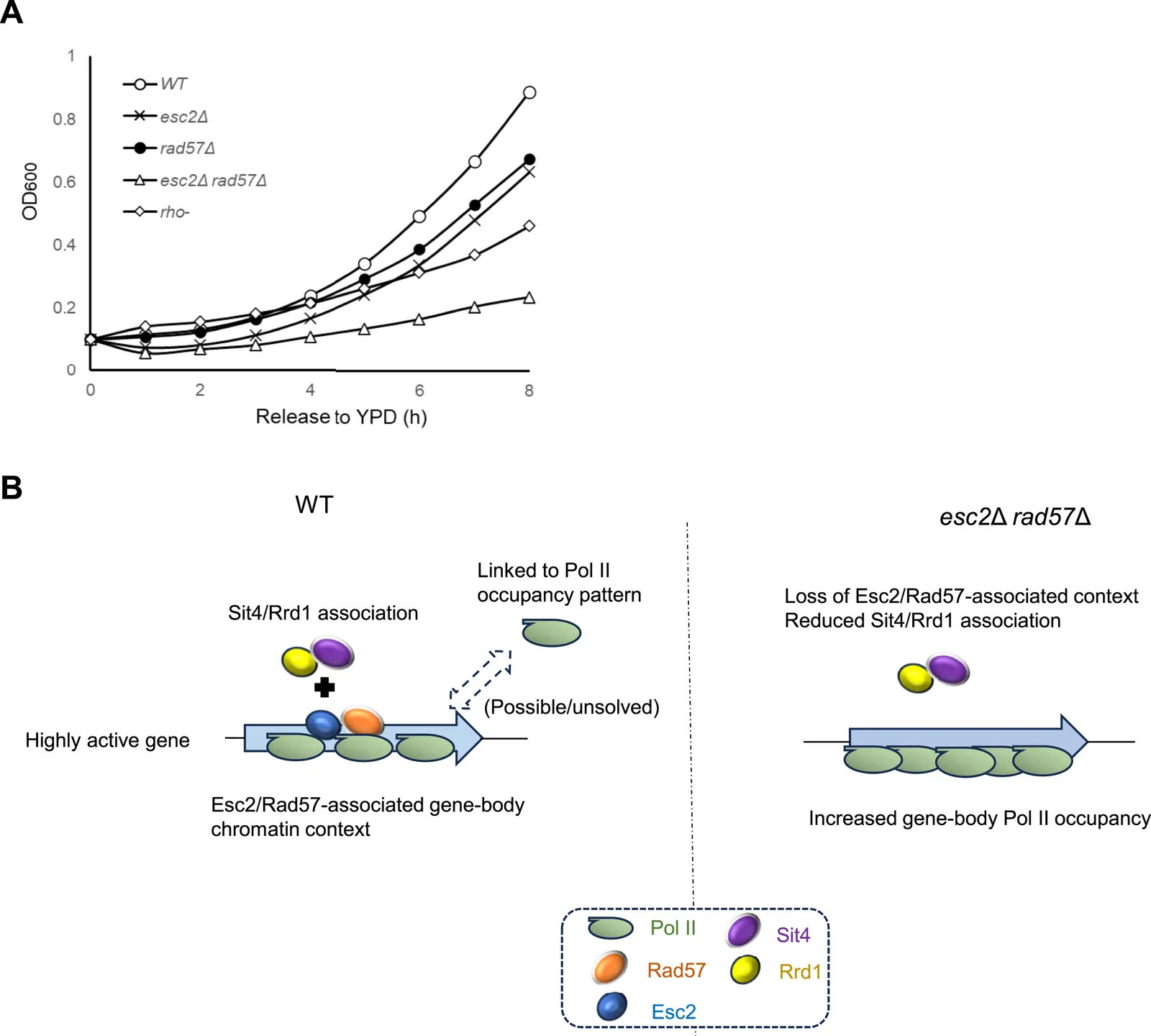
Chronic loss of Esc2/Rad57 is associated with impaired growth and a working model for altered Pol II distribution. **(A)** Growth curves of WT, *esc2*Δ, *rad57*Δ, *esc2*Δ *rad57*Δ, and rho^−^ cells cultured in YPD at 25°C. Data are shown as the mean ± SD from three biological replicates. The *esc2*Δ *rad57*Δ cell exhibits a pronounced growth defect compared with WT and single mutants. **(B)** Working model summarizing the association between Esc2/Rad57/Sit4/Rrd1 and gene-body Pol II occupancy at highly transcribed genes. In WT cells, Esc2, Rad57, Sit4, and Rrd1 are associated with a gene-body chromatin context. In *esc2*Δ *rad57*Δ cells, Sit4/Rrd1 association at representative loci is reduced, and Pol II occupancy is increased across gene bodies. The dashed double-headed arrow indicates a possible functional or chromatin-state relationship and does not imply a demonstrated direct biochemical interaction or Pol II release. The model does not imply direct Pol II eviction or release, altered transcriptional output, or a causal link to growth phenotypes.

Because *esc2*Δ *rad57*Δ cells exhibited mtDNA loss and a *rho*^−^-like phenotype, we next asked whether these broader physiological abnormalities were accompanied by impaired proliferation. Whereas *esc2*Δ and *rad57*Δ single mutants displayed only modest growth impairment, the *esc2*Δ *rad57*Δ double mutant exhibited a marked growth delay (Fig 5A). Thus, chronic combined loss of Esc2 and Rad57 is associated with impaired proliferation. However, because this phenotype likely reflects multiple contributing factors, including the transcription-associated changes described above, as well as additional physiological abnormalities, we do not interpret slow growth as a direct or exclusive consequence of altered Pol II occupancy.

### Anchor-away depletion of Rad57 does not fully recapitulate the chronic *esc2*Δ *rad57*Δ transcriptome phenotype

**Figure S7.**
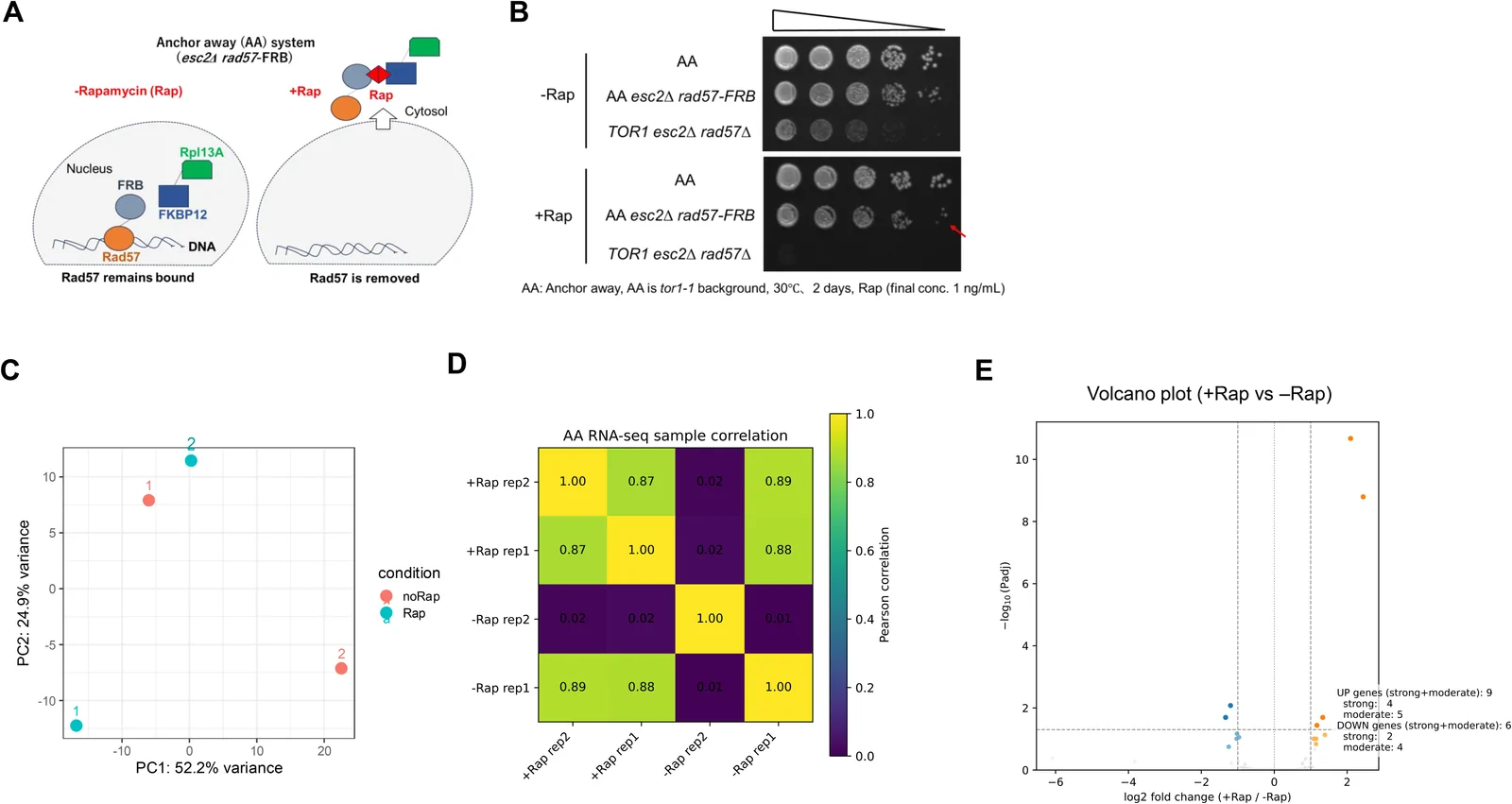
Acute nuclear depletion of Rad57 in an *esc2*Δ background. **(A)** Schematic of the Rad57-FRB anchor-away system used in an *esc2*Δ background. Rap induces cytoplasmic relocalization and nuclear depletion of Rad57-FRB (Haruki et al, 2008). **(B)** Growth assay comparing the *esc2*Δ *rad57*-*FRB* anchor-away strain and the corresponding *TOR1 esc2*Δ *rad57*Δ control strain with or without Rap. Rap treatment caused only a mild growth defect in the anchor-away strain under the tested conditions. **(C)** PCA of RNA-seq samples from −Rap and +Rap conditions. **(D)** Sample-wise Pearson’s correlation heatmap of the RNA-seq samples shown in **(C)**. One −Rap replicate showed markedly reduced correlation with the other samples, indicating appreciable replicate variability. Therefore, the anchor-away RNA-seq dataset was interpreted cautiously and was not used to draw strong quantitative or mechanistic conclusions from differential expression analysis. **(E)** Volcano plot of differential gene expression in +Rap versus −Rap conditions. The analysis did not reveal broad transcriptome-wide changes comparable to those observed in the stable *esc2*Δ *rad57*Δ mutant under the tested conditions, but this comparison should be interpreted cautiously because of the replicate variability shown in (C) and (D).

To compare the effects of chronic gene deletion with acute perturbation, we used the anchor-away system (Haruki et al, 2008) to deplete Rad57 from the nucleus in *esc2*Δ *rad57-FRB* cells (Fig S7A). In this system, rapamycin (Rap) induces association of FRB-tagged Rad57 with an FKBP12-tagged ribosomal anchor, resulting in cytoplasmic relocalization and nuclear depletion of Rad57. Rap treatment produced only a mild growth defect compared with the pronounced phenotype of the stable *esc2*Δ *rad57*Δ mutant (Fig S7B). RNA-seq analysis performed with or without Rap did not reveal broad transcriptome-wide changes comparable to those observed in the stable *esc2*Δ *rad57*Δ mutant under the tested conditions (Fig S7E). The genome-wide expression changes observed with or without Rap treatment, together with associated GO enrichment analyses, are summarized in Table S4. Principal component and Pearson’s correlation analyses further indicated appreciable replicate variability, including one −Rap sample with markedly reduced correlation to the other samples (Fig S7C and D). We therefore interpret this dataset cautiously and do not use it to draw a strong quantitative or mechanistic conclusion. Rather than viewing the weak overlap between the anchor-away transcriptome and the chronic *esc2*Δ *rad57*Δ phenotype as a mechanistic contradiction, we conclude more conservatively that acute Rad57 depletion in an *esc2*Δ background does not fully phenocopy the transcriptome changes observed in the stable *esc2*Δ *rad57*Δ mutant.

Table S4. Summary of genome-wide expression changes with or without Rap treatment and GO enrichment analyses (related to Fig S7).

## Discussion

In this study, we uncover an unexpected link between HR-related factors and transcription-associated chromatin features within gene bodies. Esc2 and Rad57 preferentially associate with the gene bodies of highly transcribed genes, and combined loss of these factors is accompanied by increased Pol II occupancy across a subset of these loci. Importantly, increased gene-body Pol II occupancy in *esc2*Δ *rad57*Δ cells does not uniformly scale with steady-state RNA output, indicating that altered Pol II occupancy within gene bodies is not equivalent to a simple global increase in productive transcription. Together, our data reveal an association between Esc2/Rad57, gene-body Pol II occupancy, and transcription-associated chromatin states at highly active genes.

A key feature of our findings is that gene-body association of Esc2 and Rad57 is not shared by the canonical HR factors Rad51 and Rad55, indicating functional specialization within the HR protein network. This distinction suggests that Esc2 and Rad57 are associated with chromatin features linked to high transcriptional load, rather than simply acting as generic HR factors at these loci. Identifying the molecular determinants that recruit Esc2 and Rad57 to active gene bodies, and clarifying how these factors distinguish transcription-associated chromatin from canonical DNA damage sites will be important topics for future investigation.

Our genome-wide and locus-specific analyses further connect Esc2 and Rad57 to the protein phosphatase Sit4 and its cofactor Rrd1. Sit4 and Rrd1 are associated with the same highly transcribed gene-body regions as Esc2 and Rad57, and ChIP-qPCR analyses show that their association at representative loci is reduced in *esc2*Δ *rad57*Δ cells. In contrast, Esc2 and Rad57 binding is not markedly reduced in *sit4*Δ cells. These results suggest that Sit4/Rrd1 association at these loci is sensitive to the Esc2/Rad57-associated chromatin context. At the same time, the weaker and less sharply gene body–centered pattern of Rrd1 suggests that its chromatin engagement may be more limited or context-dependent than that of Esc2, Rad57, and Sit4.

Because the ChIP-seq experiments were not performed with exogenous spike-in material, we cannot rigorously compare absolute Pol II ChIP-seq signal levels between WT and *esc2*Δ *rad57*Δ cells. We therefore interpret the Pol II ChIP-seq data primarily in terms of relative gene-body occupancy patterns rather than absolute genome-wide differences in Pol II abundance. The increased Pol II occupancy observed in *esc2*Δ *rad57*Δ cells does not, by itself, define the biochemical state of the polymerase. Thus, our data do not distinguish among possibilities such as slow-moving, stalled, or otherwise nonproductively retained Pol II. We therefore interpret the observed Pol II increase primarily as altered polymerase occupancy at gene bodies, while leaving open the precise mechanistic state of the retained polymerase. In this context, the anchor-away experiment is informative but should be interpreted cautiously. Under the tested conditions, acute nuclear depletion of Rad57 in an *esc2*Δ background did not reveal broad transcriptome-wide changes comparable to those observed in the stable *esc2*Δ *rad57*Δ mutant. However, because this dataset showed appreciable replicate variability, including one −Rap sample with markedly reduced correlation to the other samples, we do not use it to draw a strong quantitative or mechanistic conclusion. Rather, these data indicate that acute Rad57 depletion under the tested conditions did not fully recapitulate the chronic *esc2*Δ *rad57*Δ transcriptome phenotype, suggesting that stable loss of Esc2/Rad57 may involve cumulative and/or indirect consequences.

Finally, *esc2*Δ *rad57*Δ cells exhibit mtDNA loss, reduced mitochondrial transcripts, and slow growth. Because neither Esc2 nor Rad57 shows detectable association with mtDNA, these mitochondrial phenotypes are unlikely to reflect a direct transcriptional role at mitochondrial loci and instead most likely represent secondary consequences of combined Esc2/Rad57 deficiency. The slow-growth phenotype of *esc2*Δ *rad57*Δ cells is therefore likely to be multifactorial. In addition to the transcription-related defects described here, mitochondrial dysfunction and other genome maintenance–related abnormalities may contribute substantially to the physiological consequences of the double mutation.

Based on these observations, we propose a working model in which Esc2, Rad57, Sit4, and Rrd1 are associated with a gene-body chromatin context at highly transcribed genes that is linked to altered Pol II occupancy when Esc2 and Rad57 are lost (Fig 5B). Our data do not establish the biochemical state of the retained Pol II, nor do they demonstrate direct Pol II eviction or release in this chromatin context. They also do not exclude the involvement of additional Sit4-dependent or Sit4-independent mechanisms. Previous studies have implicated Rrd1 in the regulation of chromatin-associated Pol II (Jouvet et al, 2010; Poschmann et al, 2011). In the present study, Sit4 and Rrd1 were associated with highly transcribed gene bodies that also showed Esc2/Rad57 association, and ChIP-qPCR analysis indicated that Sit4/Rrd1 association at representative loci was reduced in *esc2*Δ *rad57*Δ cells. These observations are consistent with a possible connection between Sit4/Rrd1 association and chromatin-associated Pol II at highly transcribed genes. However, our in vivo data do not directly demonstrate Pol II eviction or release by Rrd1, and the biochemical state of the retained Pol II in *esc2*Δ *rad57*Δ cells remains unresolved. Recent studies have described global mechanisms by which transcriptional machinery, including Pol II, becomes concentrated at highly active regulatory regions such as transcriptional condensates or transcriptional hubs (Cho et al, 2018; Sabari et al, 2018; Javed et al, 2022; Lyons et al, 2025). Although we do not directly test condensate-based mechanisms here, our data suggest that gene-body chromatin features associated with Esc2/Rad57/Sit4/Rrd1 are linked to Pol II occupancy patterns at highly active genes in budding yeast. Clarifying whether and how these factors influence Pol II kinetics, chromatin association, and factor dynamics will be an important goal of future work.

## Materials and Methods

### Strains and media

Yeast strains, plasmids, and primers used in this study are listed in Table S5.

Table S5. Yeast strains, plasmids, and primers used in this study.

The parental *S. cerevisiae* strain was BY4742. Gene deletion mutants were obtained from the haploid yeast open-reading-frame deletion collection (GE Dharmacon). Cells were cultured at 30°C in YPD medium (1% yeast extract, 2% peptone, 2% glucose) or synthetic complete medium (Amberg et al, 2005). *rho*^*-*^ strains lacking mtDNA were generated by growth in YPD containing 25 μg/ml ethidium bromide and verified by growth deficiency on YPG (3% glycerol) and DAPI staining (Dirick et al, 2014). For anchor-away RNA-seq experiments, *esc2*Δ *rad57-FRB* cells were cultured in YPD at 25°C to a mid-log phase. Cultures were divided into −Rap and +Rap conditions, and Rap (Fujifilm) was added to the +Rap cultures at a final concentration of 1 ng/ml. After 2 h of treatment, cells were collected for total RNA extraction. *Escherichia coli* DH5α was used for plasmid propagation (Hanahan, 1983).

### Yeast transformation and plasmid integration

Yeast transformation was performed using the lithium acetate/PEG method (Chen et al, 1992). Integration plasmids (0.5–1.0 μg) were linearized with Bsu36I and introduced into yeast cells by heat shock at 42°C for 1 h. Transformants were selected on appropriate synthetic complete dropout media and verified by PCR and immunoblotting (Chen et al, 1992).

### Synthetic-lethal screen

The parental strain HMY1770 (MATα *his3*Δ *leu2*Δ*1 met15*Δ*0 ura3*Δ*0 rad57*Δ*::kanMX esc2*Δ*::sphis5+* + p*URA3-RAD57*) was mutagenized with ethyl methanesulfonate as previously described (Amberg et al, 2005).

Approximately 18,000 colonies were screened for 5-FOA sensitivity, yielding 62 candidates. Transformable clones (n = 26) were retested and screened for complementation using the *S. cerevisiae* YEp13 genomic library (NBRP, Japan). Insert sequences were recovered in *E. coli* and identified by Sanger sequencing.

### Chromatin immunoprecipitation

ChIP was performed as previously described (Lee & Keung, 2018; Masumoto et al, 2024) with minor modifications.

Log-phase cultures (OD_600_ = 0.5–0.6) were cross-linked with 1% formaldehyde for 15 min, quenched with glycine, and lysed with glass beads. Chromatin was sonicated to an average fragment size of ∼500 bp and immunoprecipitated using anti-FLAG M2 affinity gel (Cat. A2220; *Sigma-Aldrich*) or anti-RNA Pol II antibody (Cat. 102660; *Active Motif*, RRID:AB_2732926). Crosslinks were reversed at 65°C overnight, and DNA was purified by phenol–chloroform extraction and ethanol precipitation.

### ChIP-qPCR

ChIP-qPCR was performed using LightCycler 480 SYBR Green I Master (Cat. 04707516001; Roche Life Science) for the experiments shown in Fig 4F and G, and THUNDERBIRD Next SYBR qPCR Mix (Cat. QPX-201; Toyobo) for the experiments shown in Fig 3G. Percent input values were calculated as described previously (Haring et al, 2007), and are shown without subtraction of no-antibody or no-tag controls. All experiments were performed with three independent biological replicates unless otherwise indicated. Primer sequences are listed in Table S5. Statistical tests used for each experiment are indicated in the corresponding figure legends.

### ChIP-seq library preparation and analysis

ChIP-seq libraries were prepared using NEBNext ChIP-Seq Kit or Accel-NGS 1S Plus DNA Kit and sequenced on an Illumina NovaSeq 6000 platform (150 bp paired-end). Reads were trimmed with Trim Galore! and aligned to the *S. cerevisiae* sacCer3 genome using Bowtie2. Coverage tracks were generated as bigWig files using deepTools bamCoverage with CPM normalization and a bin size of 10 bp. For Flag-tagged factor ChIP-seq heatmaps, subtraction of the common no-tag IP control was performed using CPM-normalized bigWig files, not raw read counts, before visualization. For Pol II ChIP-seq browser views, no-antibody IP control signals were likewise subtracted using CPM-normalized bigWig files. Genome-wide heatmaps and metagene profiles were generated using computeMatrix and plotHeatmap aligned relative to transcription start and end sites. Gene clustering and GO enrichment analyses were performed in Python and g:Profiler (FDR < 0.05). All experiments were performed with two independent biological replicates. The unsubtracted no-tag IP ChIP-seq signal was also visualized over the same gene set and gene order as the Flag-tagged factor heatmaps as a background control. Sequencing depth and normalization information for the ChIP-seq datasets is summarized in Table S6.

Table S6. Summary of ChIP-seq and RNA-seq data.

### RNA-seq

Total RNA was extracted using RNeasy Mini Kit (Cat. 74104; Qiagen). Libraries were prepared after rRNA depletion or poly**(A)** selection as indicated and sequenced on an Illumina NovaSeq 6000. Reads were aligned to sacCer3 using HISAT2 or Bowtie2. Gene counts were obtained with featureCounts and analyzed using DESeq2 (Love et al, 2014).

Genes with adjusted *P* < 0.05 were considered differentially expressed. PCA, correlation analyses, and visualization were performed in R. All experiments were performed with two independent biological replicates.

### RNA RT–qPCR

RNA RT–qPCR was performed using One-Step PrimeScript RT–PCR Kit (Perfect Real Time) (Cat. RR064A; Takara Bio). All experiments were performed with three independent biological replicates. Primer sequences are listed in Table S5.

### mtDNA detection

Cells were stained with DAPI and MitoTracker Red CMXRos. Fluorescence images were acquired using a ZEISS LSM 800 confocal laser-scanning microscope equipped with an Airyscan detection module (Carl Zeiss) and a 63×/1.40 NA oil-immersion objective. DAPI was excited with a 405-nm laser, and emission was collected over 400–499 nm. MitoTracker Red was excited with a 561-nm laser, and emission was collected over 565–650 nm. Images were acquired using identical acquisition settings within each experiment and processed equivalently for comparison.

### Statistical analysis of lifespan

Statistical details, including sample size and tests used, are provided in figure legends. Error bars represent standard deviation from independent biological replicates. Statistical analyses were performed using R, with *P* < 0.05 considered significant.

Table S7. NGS datasets.

## Supplementary Material

Reviewer comments

## Data Availability

NGS datasets generated in this study have been deposited in the DNA Data Bank of Japan and are summarized in Table S7 (accession numbers: DRA012865, DRA019921, DRA019968, DRA019969, DRA019971, DRA020301, and DRA023644).
