# Supplementary material for: Esc2 and Rad57 contribute to RNA polymerase II distribution at highly transcribed genes
Source: Life Sci Alliance. 2026 Oct 12;10(1):e202603673. doi: 10.26508/lsa.202603673 (PMC13658948; doi:10.26508/lsa.202603673)
Supplement: Supplementary file 1 [file LSA-2026-03673_review_history.pdf]

Esc2 and Rad57 contribute to RNA polymerase II distribution at highly transcribed genes

Hiroshi Masumoto, Hideki Muto, Yohei Kurosaki, and Hironori Niki
DOI: https://doi.org/10.26508/lsa.202603673

Corresponding author(s): Hiroshi Masumoto, Nagasaki University

Review Timeline:

Submission Date:	2026-02-17
Editorial Decision:	2026-04-05
Revision Received:	2026-06-08
Editorial Decision:	2026-07-07
Revision Received:	2026-07-31
Editorial Decision:	2026-09-17
Revision Received:	2026-09-24
Accepted:	2026-09-30

Scientific Editor: Sarita Hebbar

Transaction Report:

(Note: With the exception of the correction of typographical or spelling errors that could be a source of ambiguity, letters and reports are not edited. The original formatting of letters and referee reports may not be reflected in this compilation.)

April 5, 2026

Re: Life Science Alliance manuscript #LSA-2026-03673-T

Dr. Hiroshi Masumoto
Nagasaki University School of Medicine
Biomedical Research Support Center (BRSC), Research Center for Advanced Genomics
Japan

Dear Dr. Masumoto,

Thank you for submitting your manuscript entitled "Esc2 and Rad57 contribute to RNA polymerase II distribution at highly transcribed genes" to Life Science Alliance. The manuscript was assessed by three expert reviewers, whose comments are appended to this letter.

As you will read Reviewers 2 and 3 find this observation, on the enrichment of recombination-related factors at certain loci, interesting and of potential value. That said, all the reviewers are in agreement that the manuscript in its current form requires more accurate evidence to substantiate your major claims. We therefore invite you to submit a revised manuscript addressing their concerns.

We agree with the reviewers that you need to provide evidence to substantiate a direct link between Esc2/Rad57 localisation, Pol II dynamics, and the effect on general transcription regulation. We point you to the suggestions on data analyses
-from Rev 1 (point 2) and Rev 2 (point 1) in comparing chromatin occupancy of Pol II, recombination factors and transcription.
-from Rev 2 (point 5) and Rev 3(Figure 3F-G) for double mutant analyses of link between recombination factors and Pol II.
In this connection, Reviewer 1 (point 2) suggested direct measurement of newly synthesised RNAs but we agree that this is not required in a revised version. We ask that you nevertheless propose a mechanism underlying the connection between these recombination factors and Pol II dynamics in transcription.

We bring your attention to technical concerns raised by Rev 1, points 3-4 and Rev 3, additional comment 2 (ChipSeq experiments) and Rev2, points 2-3 (RNASeq data and analyses) and agree that you must resolve these concerns.

Finally we concur with all the reviewers that you must acknowledge other contributing factors whilst discussing the growth phenotype in the mutants. We noted that you have partly attributed the growth phenotypes to mtDNA defects on page 14. This must be accompanied by moderation of claims related to cell proliferation in the context of the mutants on page 13.

When submitting the revision, please include a letter addressing the reviewers' comments point by point. While a rebuttal must respond to all points in some form, additional experiments to resolve these points, other than indicated above, are not required.

I would be happy to discuss the revision in more detail via email or phone/videoconferencing. Please let me know which option you prefer, if any.

To upload the revised version of your manuscript, please log in to your account: https://lsa.msubmit.net/cgi-bin/main.plex

You will be guided to complete the submission of your revised manuscript and to fill in all necessary information. Please get in touch in case you do not know or remember your login name.

The typical timeframe for revisions is three months. Please note that papers are generally considered through only one revision cycle, so strong support from the referees on the revised version is needed for acceptance.

While you are revising your manuscript, please also attend to the below editorial points to help expedite the publication of your manuscript. Please direct any editorial questions to the journal office.

Thank you for this interesting contribution to Life Science Alliance. We hope that the comments below will prove constructive as your work progresses, and we are looking forward to receiving your revised manuscript.

Sincerely,

Sarita Hebbar, PhD
Scientific Editor
Life Science Alliance
http://www.lsajournal.org

A. THESE ITEMS ARE REQUIRED FOR REVISIONS

-- A letter addressing the reviewers' comments point by point.

-- An editable version of the final text (.DOC or .DOCX) is needed for copyediting (no PDFs).

-- High-resolution figure, supplementary figure and video files uploaded as individual files: See our detailed guidelines for preparing your production-ready images, https://www.life-science-alliance.org/authors

-- Summary blurb (enter in submission system): A short text summarizing in a single sentence the study (max. 200 characters including spaces). This text is used in conjunction with the titles of papers, hence should be informative and complementary to the title and running title. It should describe the context and significance of the findings for a general readership; it should be written in the present tense and refer to the work in the third person. Author names should not be mentioned.

-- By submitting a revision, you attest that you are aware of our payment policies found here: https://www.life-science-alliance.org/copyright-license-fee

B. MANUSCRIPT ORGANIZATION AND FORMATTING:

Full guidelines are available on our Instructions for Authors page, https://www.life-science-alliance.org/authors

We encourage our authors to provide original source data, particularly uncropped/-processed electrophoretic blots and spreadsheets for the main figures of the manuscript. If you would like to add source data, we would welcome one PDF/Excel-file per figure for this information. These files will be linked as supplementary "Source Data" files.

IMPORTANT: It is Life Science Alliance policy that if requested, original data images must be made available. Failure to provide original images upon request will result in unavoidable delays in publication. Please ensure that you have access to all original microscopy and blot data images before submitting your revision.

Reviewer #1 (Comments to the Authors (Required)):

In this manuscript, the authors propose that homologous-recombination-related factors Esc2 and Rad57 are enriched on highly-transcribed genes and that their loss can modulate RNA polymerase II (Pol II) distribution. This unexpected link remains to be confirmed with more convincing results. Moreover, potential mechanisms are unclear and were not proposed. Several major concerns need to be addressed and, in its current form, the work seems preliminary.

Major concerns:
1. No arguments are presented that Esc2 and Rad57 can play an active role in Pol II dynamics and a potential mechanism remains to be proposed.

2. No direct evidence is provided that Pol II accumulates without changes in transcriptional activity. Pol II ChIP and RNAseq should be precisely compared and completed with experiments for a direct measurement of newly synthetized RNAs (metabolic-labelling approach) to show if the transcriptional activity is modified or not.

3. The specificity of Esc2 and Rad57 ChIP enrichment on highly transcribed genes need to be confirmed. It is known for many years that highly-transcribed genes can present an artificial ChIP enrichment and untagged strain or no antibody controls are essential (for example, Teytelman et al PNAS 2013, Park et al. PLoS One 2013 and Jeronimo & Robert NSMB 2014). These essential controls are missing and they are needed to conclude on the specific ChIP enrichment. The lower signals with Rad51 and Rad55 can have many reasons, for example that ChIP does not work well for these proteins. Figure 1 should contain an untagged or mock IP control. Moreover, it should be tested if the presence of Esc2 and Rad57 on chromatin is dependent on transcription by performing ChIP with internal (spike-in) normalisation in conditions that effected transcription.

4. An appropriated normalization of ChIPseq data such as spike-in normalization is needed to compare different samples in a correct way and to conclude about changes between strains.

5. The link between growth phenotypes of esc2Δ rad57Δ and ChIPseq results can be indirect. The loss of mitochondrial DNA as the major effect in esc2Δ rad57Δ could not be excluded.

6. The proportion of genes with both increased RNA level in esc2Δ rad57Δ and Esc2/Rad57 ChIP signals is very low, only a few

dozen of genes (Fig. S2F), similar to very low proportion of genes with both increased Pol II level in esc2Δ rad57Δ and Esc2/Rad57 ChIP signals (Fig 2C, 41 genes). It is difficult to make conclusions with this low proportions.

7. Genome-wide data should be available on public databases accessible for different countries, and the accession numbers should be provided.

Other concerns:
8. The organization of results in small numbered parts composed of only one paragraph should be modified to regroup them into more structured results.

9. For data analysis, the number of replicates should be clearly indicated and the way how replicates were taken into account in analysis and the comparison between the replicates should be systematically presented.

10. Introduction, p. 3 - a recent paper on promoter-proximal pausing across evolution should be cited (Chivu NSMB 2026) and the text of introduction should be modified accordingly.

11. Figure S7D - to comment on the scale of the graph, the signal is not flat.

12. The scale in Fig S7D should be indicated.

13. There is a problem with the text font on page 21 (additional information, author contribution), page 26 (Fig. 1 legend), page 29 (Figure S1 legend).

14. To correct "gene-wise" to "genome-wide" in Table S2, 3, 4, 5 titles.

15. Figure S1D - problem with headings, Figure S2E - display problem.

Reviewer #2 (Comments to the Authors (Required)):

This manuscript by Hiroshi Masumoto et al. investigates the roles of Esc2 and Rad57 in modulating RNA polymerase II (Pol II) distribution at highly transcribed genes. The authors demonstrate that these recombination-related factors associate with the gene bodies of housekeeping genes. Notably, the loss of Esc2 and Rad57 results in increased Pol II occupancy within these regions. While the discovery that recombination factors may perform functions beyond DNA repair is novel and compelling, the current data presentation hinders interpretation. Specifically, the relationship between the recruitment sites of these proteins and the resulting changes in transcription levels or Pol II occupancy remains unclear. Rather than relying primarily on gene clustering approaches, the authors should provide a direct gene-by-gene correlation to substantiate the functional impact of Esc2/Rad57 recruitment on Pol II dynamics and transcriptional output. Furthermore, the rationale for performing the primary analysis on the esc2Δ rad57Δ double mutant is not fully justified by the data presented. Finally, the cell death phenotype described in the final figure lacks a clear mechanistic connection to the proposed function of these genes in transcriptional regulation.

To substantiate the claims made in the manuscript, the authors must establish a more direct link between Esc2/Rad57 chromatin localization and transcriptional control, rather than relying solely on indirect correlations. Below, I outline specific technical concerns and requested analyses that are necessary to address these gaps.

Major Points:

1. The authors categorize genes into groups (C1-C3) based on Esc2/Rad57 signal intensity and note that C1 genes are enriched for pathways typically associated with high expression, such as ribosome biogenesis. However, this functional enrichment is an indirect proxy for transcriptional activity. To establish a more robust connection, the authors should move beyond categorical clustering and perform a direct gene-by-gene correlation analysis. Specifically, the authors should:

a) Perform a genome-wide correlation analysis between Esc2/Rad57 chromatin occupancy and mRNA levels.
b) Provide metagene profiles of Esc2/Rad57 binding across gene cohorts categorized by expression level.

These analyses would clarify whether the enrichment observed at specific highly transcribed genes, such as TDH3 and CDC19 (Figure 1A), represents a general feature of highly transcribed genes bound by Esc2/Rad57, or whether these examples represent isolated cases.

2. There are significant concerns regarding the quality and presentation of the RNA-seq data (Figure S2). While the esc2Δ rad57Δ double mutant volcano plot displays the expected "cloud" distribution of the yeast transcriptome, the single mutant plots appear to be missing the vast majority of genes. Even if the single mutants produce only modest transcriptome changes, the volcano plot should represent the entire detected transcriptome in order to demonstrate that the experiment was technically successful and to provide a stable baseline for comparison. The absence of these data points suggests either a technical issue

during library preparation for the single mutants or inappropriate filtering of the dataset that prevents fair comparison with the double mutant.

Furthermore, the PCA analysis shows high variance between the WT replicates and between the esc2 single mutant replicates (Figure S2D). This variability is concerning. The authors should address these discrepancies to substantiate their claim that transcriptional defects are unique to the esc2Δ rad57Δ double mutant.

3. In Figure S2, the authors claim that upregulated genes in the esc2Δ rad57Δ mutant are enriched in Clusters 1 and 2. However, the actual overlap appears to be very limited. Only 12 genes out of 286 upregulated genes overlap with Cluster 1. The authors should clarify why the vast majority of genes bound by these factors show no detectable transcriptional change upon deletion.

4. The authors report that acute Rad57 depletion using an anchor-away system in an esc2Δ background does not recapitulate the transcriptome alterations observed in the stable double-deletion mutant. However, the variance between biological replicates in Figure S4C appears to be very high. This suggests that the sequencing data may be noisy or affected by batch effects, which could strongly influence downstream differential expression analysis. High intra-condition variance reduces statistical power and may explain the sparse results observed in the volcano plot shown in Figure S4D. The authors should provide a Pearson correlation matrix for all RNA-seq samples to quantify replicate consistency. In addition, the authors should clarify why the volcano plots display such a limited number of genes.

5. In Figure 2, to clarify the relationship between these recombination factors and Pol II dynamics, the authors should perform a direct gene-by-gene correlation between Esc2/Rad57 recruitment levels (ChIP-seq signal) and Pol II occupancy in the esc2Δ rad57Δ mutant.

6. The authors fail to establish a causal link between the growth defects shown in Figure 4 and the reported transcriptional changes. Including "slow growth" in the final model without a clear mechanistic explanation is therefore speculative. As presented, the growth phenotype could reflect secondary consequences of genomic instability, recombination defects, or broader cellular stress rather than a direct transcriptional effect of Esc2 and Rad57 loss.

Minor points:

1. The manuscript lacks a clear rationale for specifically investigating the association of Esc2 and Rad57 with chromatin. Without a clearly stated hypothesis or preliminary observation explaining why these particular recombination factors were selected, it is difficult for the reader to identify the central question motivating the study.

2. The authors should briefly explain the mechanism of the anchor-away system and clarify the rationale for using rapamycin to induce nuclear depletion.

Reviewer #3 (Comments to the Authors (Required)):

In this study by Masumoto et al., the authors show that recombination-related factors, Esc2 and Rad57, are enriched in the coding regions of many highly expressed genes, whereas Rad51 and Rad55 are enriched to a lesser extent. In the esc2/rad57 double mutant, the authors note hyper-enrichment of Pol II in ORFs. However, nuclear depletion of Rad57 in the esc2 background did not yield the same phenotype as that of the double-deletion mutant. The authors suggest that the phenotype observed in the double mutant might result from prolonged loss of these factors. The authors also find that the downregulated genes in the double mutant were enriched for the genes involved in mitochondrial function. The authors note that this was likely due to mitochondrial loss in the double mutant.
Although the authors' finding that recombination-related factors are enriched in coding regions is interesting, several issues remain unresolved. A key unresolved question is whether the enrichment of Esc2 and Rad57 in ORFs, along with increased Pol II occupancy in coding regions in the double mutant, has physiological significance. Although the authors report growth defects in the double mutant, these may result from disruptions in other functions. The second unresolved issue concerns what the increase in Pol II levels indicates regarding transcription activity. Generally, higher Pol II levels in ORFs correlate with higher transcriptional activity and involve more efficient initiation. However, this is not reflected in RNA-seq data, and the increase in Pol II poorly correlates with mRNA production; although this could be due to differences in the assay used (ChIP-seq versus RNA-seq). Alternatively, it could suggest stalled or slow-elongating Pol II. And if the increased Pol II accumulation signifies elongation defects, then the genes with massive accumulation should show transcription defects in the mutant, but this does not seem to be the case either. The authors provide data that Sit4 and Rrd1 enrichment is dependent on Esc2 and Rad57. Because this paper does not establish a clear role for Esc2 and Rad57 enrichment in ORFs, conducting genome-wide ChIP-seq for Sit4 and Rdr1 could help in implicating their function in regulating Sit4 and Rdr1 occupancies.
Additional comments:
o Authors should also show protein levels for the recombination-related factors to ensure that the low enrichment of Rad51 and Rad55 is not due to their lower expression relative to Rad57 and Esc2.

o ORFs of several highly expressed genes are classified as hyper-ChIP-able regions. The authors identify 41 genes that overlap between Esc2-Rad57 and Pol II cluster 1 (Fig. 2C). Do the authors observe statistically significant enrichment when the hyper-ChIP-able regions are removed?
o The authors should show Pol II occupancy by ChIP-qPCR for at least a couple of genes that reveal high Pol II ORF occupancy in the double mutant, and also RNA levels for those genes by qRT-PCR.
o The authors find that Esc2, Rad57, and Sit4 are enriched in ORFs and present the enrichment as heatmaps (Fig. 3A). Are the genes sorted in the same order, or are they sorted by their individual enrichment levels? It would be important to show a correlation plot for Pol II and the three factors' ORF occupancies.
o Fig. 3C shows heatmaps that reveal 39 genes enriched across all four factors. Is this significant?
o Fig 3F-G. The authors should show Pol II levels at these genes.
o The authors propose that Rad57/Esc2 prevents hyperaccumulation of Pol II. If that is the case, one would observe a significant increase in mRNA and Pol II levels at the Rad57/Esc2-enriched genes in the double mutant.
o The authors use C1, C2, and C3 clusters to define Pol II occupancy (Fig 2B) and same nomenclature for the occupancies of Esc2 and Rad57. It would be easier for the reader if they named clusters as Pol II-C1 cluster or Esc2-C1 cluster.

Dear Prof. Sarita Hebbar and Reviewers,

We thank the reviewers and editor for their constructive comments. In response, we substantially revised the manuscript to strengthen the direct relationship between Esc2/Rad57 chromatin localization, Pol II accumulation, and gene-selective steady-state RNA output. Specifically, we added no-tag control analyses, direct genome-wide correlation analyses, locus-specific Pol II ChIP-qPCR and RT-qPCR validation, and we moderated the interpretation of growth-related phenotypes. These revisions address the editor's central concerns regarding mechanistic linkage, technical rigor, and overstatement of physiological consequences.

Reviewer #1

We thank the reviewer for the careful and constructive evaluation of our manuscript. We appreciate the reviewer's concerns regarding the mechanistic interpretation, the specificity of the ChIP-seq signals, and the need for a more direct comparison between Pol II occupancy and transcriptional output. In the revised manuscript, we have substantially strengthened the study by adding no-tag control analyses, direct genome-wide comparative analyses, locus-specific Pol II ChIP-qPCR and RT-qPCR validation, and by moderating claims that were previously stated too strongly. As outlined below, we believe these revisions directly address the reviewer's major concerns.

Major concern 1

Reviewer comment:

No arguments are presented that Esc2 and Rad57 can play an active role in Pol II dynamics and a potential mechanism remains to be proposed.

Response:

We agree with the reviewer that the previous version did not sufficiently articulate how Esc2 and Rad57 could contribute actively to Pol II dynamics. In the revised manuscript, we have addressed this point in two ways. First, we now provide additional locus-specific validation showing that Pol II occupancy increases at representative target genes in *esc2*Δ *rad57*Δ cells, supporting the idea that Esc2 and Rad57 normally act to restrain excess Pol II accumulation on gene bodies rather than merely correlating with transcriptional activity. Second, we have revised the Discussion to present a more explicit but appropriately cautious working model. In this model, Esc2 and Rad57 are proposed to act at Pol II-rich, highly transcribed loci to promote proper Pol II distribution and prevent excessive retention of elongating polymerase, possibly in coordination with Sit4/Rrd1-dependent processes. We have also revised the manuscript throughout to distinguish more clearly between conclusions directly supported by the data and mechanistic interpretation. Accordingly,

we no longer describe Esc2/Rad57 as general transcriptional repressors, but rather as factors that contribute to transcriptional homeostasis by limiting abnormal Pol II accumulation at highly active genes.

Changes made in the revised manuscript

- Results, subsection describing Pol II accumulation at representative loci: added locus-specific Pol II ChIP-qPCR validation showing increased Pol II occupancy in *esc2*Δ *rad57*Δ cells. (**p. 9, lines 4-7**, **Figure 3G**).
- Discussion, paragraph beginning *"In this study, we uncover an unexpected link..."*: revised the mechanistic interpretation to a cautious working model in which Esc2/Rad57 limit abnormal Pol II accumulation at highly active genes. (**p. 14, lines 2-10**)
- Discussion, paragraph beginning *"Our genome-wide and locus-specific analyses further connect..."*: clarified the possible relationship between Esc2/Rad57 and Sit4/Rrd1. (**p. 14, lines 19-23**)

Major concern 2

Reviewer comment:

No direct evidence is provided that Pol II accumulates without changes in transcriptional activity. Pol II ChIP and RNAseq should be precisely compared and completed with experiments for a direct measurement of newly synthetized RNAs (metabolic-labelling approach) to show if the transcriptional activity is modified or not.

Response:

We appreciate this important point and agree that the previous version did not sufficiently distinguish Pol II accumulation from transcriptional output. In the revised manuscript, we addressed this issue by strengthening the comparison between Pol II occupancy and RNA levels at both the locus-specific and genome-wide levels. Specifically, we added Pol II ChIP-qPCR and RT-qPCR analyses at representative loci, which directly show that increased Pol II occupancy in *esc2*Δ *rad57*Δ cells is not always accompanied by a proportional increase in RNA output. We also incorporated genome-wide comparative analyses relating Pol II enrichment, Esc2/Rad57 occupancy, and RNA-seq changes, which similarly indicate that elevated Pol II accumulation does not uniformly translate into increased steady-state mRNA levels. Importantly, we have revised the manuscript text accordingly and no longer present increased Pol II occupancy as evidence for unchanged transcriptional activity per se. Instead, we now state more cautiously that abnormal Pol II accumulation in *esc2*Δ *rad57*Δ is not uniformly coupled to proportional increases in steady-state RNA abundance. Although direct measurement of newly synthesized RNA would indeed be informative, the editor's decision letter indicated that such an experiment was not required for the

revision. We therefore focused our revision on strengthening the direct comparison between Pol II occupancy and transcript output using additional locus-specific and genome-wide analyses, rather than performing metabolic labeling.

Changes made in the revised manuscript

- **Results**, subsection describing Pol II behavior in *esc2*Δ *rad57*Δ cells: added locus-specific RT-qPCR and Pol II ChIP-qPCR analyses at representative loci. (Figure 3F and 3G and corresponding legends). (**p. 9, lines 4-7**)
- **Results**, paragraph describing the genome-wide comparison between Pol II occupancy and RNA changes: added the statement that increased Pol II occupancy does not uniformly translate into proportional RNA increase "Genome-wide comparison likewise showed that large changes in Pol II occupancy…." **(p. 9, lines 7-8)**
- **Figure 3H** and corresponding legend: clarified that large Pol II changes are often associated with only modest RNA changes.
- **Discussion**, paragraph beginning *"The increased Pol II occupancy observed..."*: revised the interpretation to state that the accumulated Pol II does not necessarily reflect uniformly productive transcription. **(p. 15, lines 4-14)**

Major concern 3

Reviewer comment:

The specificity of Esc2 and Rad57 ChIP enrichment on highly transcribed genes need to be confirmed... untagged strain or no antibody controls are essential... Figure 1 should contain an untagged or mock IP control. Moreover, it should be tested if the presence of Esc2 and Rad57 on chromatin is dependent on transcription by performing ChIP with internal (spike-in) normalisation in conditions that effected transcription.

Response:

We agree that the lack of appropriate background controls was a major weakness of the original submission. In the revised manuscript, we therefore incorporated no-tag control analyses and directly compared Esc2-4F and Rad57-4F ChIP-seq signals with no-tag control signals both at representative loci and in genome-wide summary analyses. These revised analyses show that no-tag samples indeed exhibit modest enrichment over highly transcribed genes, consistent with previous observations that such loci can display elevated background in ChIP-based assays. Importantly, however, the Esc2-4F and Rad57-4F signals reproducibly exceed this background, indicating that the observed enrichments cannot be explained by no-tag background alone. We have revised the figures and manuscript text accordingly. In particular, we now explicitly

acknowledge the presence of modest background signal over highly transcribed genes and interpret Esc2/Rad57 occupancy more conservatively as enrichment above background, rather than as background-free binding. We agree that testing whether Esc2/Rad57 chromatin association is transcription-dependent using spike-in-normalized ChIP under acute transcriptional perturbation would be informative. However, because the original ChIP-seq experiments were not designed with spike-in normalization, we could not rigorously address this point retrospectively. We therefore do not make strong claims in the revised manuscript about the transcription dependence of Esc2/Rad57 recruitment per se, and instead focus our conclusions on the reproducible enrichment of Esc2/Rad57 above no-tag background at highly transcribed loci.

Changes made in the revised manuscript

- **Figure 1A** and corresponding legend: added **no-tag IP controls** to representative browser views.
- **Figure 1B** and corresponding legend: added **no-tag control** profiles to metagene analysis.
- **Results**, subsection beginning *"Esc2 and Rad57 preferentially associate..."*: explicitly state that Esc2/Rad57 signals exceed modest no-tag background at highly transcribed loci. (**p. 6, lines 4-18**)
- **Discussion**, paragraph discussing ChIP interpretation: clarified that the current data do not resolve whether Esc2/Rad57 chromatin association is directly transcription-dependent because the experiments were not performed with spike-in normalization under acute transcriptional perturbation. (**p. 14, lines 2-10**)

Major concern 4

Reviewer comment:

An appropriated normalization of ChIPseq data such as spike-in normalization is needed to compare different samples in a correct way and to conclude about changes between strains.

Response:

We agree that spike-in normalization would provide a more rigorous basis for strict quantitative comparison of ChIP-seq signal across strains. Because the original ChIP-seq datasets were not generated with exogenous spike-in material, we could not retrospectively implement this normalization strategy. In response, we adopted a more conservative interpretive framework in the revised manuscript. Specifically, we now rely primarily on (i) enrichment above no-tag background, (ii) reproducible within-dataset distribution patterns across gene classes and clusters, and (iii) independent locus-specific validation by Pol II ChIP-qPCR. At the same time, we have moderated statements that would require strict absolute cross-sample normalization and now avoid overstating conclusions about global quantitative differences in ChIP signal between strains

where spike-in normalization would be required.

Changes made in the revised manuscript

- **Results**, sections describing Esc2/Rad57 ChIP-seq analyses: revised the text to emphasize signal above no-tag background and reproducible within-dataset patterns rather than strict absolute quantitative differences between strains. (**p. 6, lines 4-8**)

Major concern 5

Reviewer comment:

The link between growth phenotypes of esc2Δ rad57Δ and ChIPseq results can be indirect. The loss of mitochondrial DNA as the major effect in esc2Δ rad57Δ could not be excluded.

Response:

We agree with the reviewer that the growth phenotype of *esc2Δ rad57Δ* cells is likely to be multifactorial and that mitochondrial dysfunction, including mtDNA instability/loss, could contribute substantially to this phenotype. In the revised manuscript, we therefore no longer present slow growth as a direct or exclusive consequence of altered Pol II distribution. Instead, we distinguish more clearly between the molecular phenotypes described in this study—such as abnormal Pol II accumulation and defective transcriptional homeostasis—and broader physiological consequences that may arise secondarily in the double mutant. We have revised the Discussion and model accordingly to acknowledge that mitochondrial dysfunction and other genome-maintenance-related defects may contribute to the observed growth defect.

Changes made in the revised manuscript

- **Results, subsection beginning *"Reduced mitochondrial transcripts in esc2Δ rad57Δ are associated with mitochondrial DNA loss"***: added analyses linking reduced mitochondrial transcripts to mtDNA loss and a rho$^-$-like phenotype. **(p. 11, lines 15-23)**
- **Results, subsection beginning *"Chronic loss of Esc2/Rad57 is associated with slow growth"*:** revised the text to state explicitly that the growth defect is not interpreted as a direct or exclusive consequence of altered Pol II distribution. **(p. 12, lines 4-12)**
- **Discussion, paragraph beginning *"Finally, esc2Δ rad57Δ cells exhibit mitochondrial DNA loss..."*:** clarified that slow growth is likely multifactorial and may reflect both transcription-related and mitochondrial defects. **(p. 15, lines 15-22)**
- **Figure 5B and corresponding legend:** revised the working model to avoid presenting growth defects as a direct single-step consequence of altered Pol II distribution.

Major concern 6

Reviewer comment:

The proportion of genes with both increased RNA level in esc2Δ rad57Δ and Esc2/Rad57 ChIP signals is very low... It is difficult to make conclusions with this low proportions.

Response:

We agree that the overlap is limited if interpreted strictly as a binary intersection between Esc2/Rad57-bound genes and genes showing significantly increased RNA levels in *esc2Δ rad57Δ* cells. In the revised manuscript, we therefore avoid relying on overlap counts alone as the primary basis for interpretation. Instead, we now emphasize cluster-level and continuous-variable analyses, which more appropriately capture the relationship between Esc2/Rad57 occupancy, Pol II behavior, and transcript output. Our revised interpretation is that Esc2/Rad57 binding marks a subset of highly active gene bodies, whereas the effect of *esc2Δ rad57Δ* on steady-state RNA accumulation is selective rather than global. Accordingly, we do not argue that most Esc2/Rad57-bound genes must necessarily display significant RNA increases upon deletion; rather, we interpret the data as indicating that Esc2/Rad57 occupancy is associated with highly active chromatin contexts in which transcriptional consequences become apparent only for a subset of genes.

Changes made in the revised manuscript

- **Results, subsection describing Esc2/Rad57-defined clusters and RNA-seq changes:** revised the interpretation to emphasize that steady-state RNA changes are enriched in ER-C1 and ER-C2 relative to ER-C3, while remaining selective rather than universal. (**p. 7, line 22-p. 8, line 2**)
- **Figure 2C legend**: revised to present the RNA-seq changes in Esc2/Rad57-defined gene classes as supportive rather than as a stand-alone basis for interpretation.
- **Results, subsection describing Pol II accumulation:** added cluster-level and genome-wide comparative analyses linking Esc2/Rad57 occupancy to Pol II behavior and selective RNA changes. (**p. 8, line 22-p. 9, line 3, Figure 3D and 3E and the legend**)

Major concern 7

Reviewer comment:

Genome-wide data should be available on public databases accessible for different countries, and the accession numbers should be provided.

Response:

We agree. The genome-wide datasets have now been deposited in DDBJ, which is publicly accessible internationally, and the corresponding accession numbers have been added to the revised manuscript, including the Data Availability section and Table S7.

Changes made in the revised manuscript

- **Data Availability section**: added the DDBJ accession numbers for all NGS datasets generated in this study. (**p. 20 lines 12-15**)
- **Table S7**: included the detailed summary of deposited datasets and corresponding accession information.

Other concern 8

Reviewer comment:

The organization of results in small numbered parts composed of only one paragraph should be modified to regroup them into more structured results.

Response:

We agree and have reorganized the Results section into more structured subsections in the revised manuscript. This revision improves the flow of the Results and aligns each section more clearly with the central biological questions addressed in the study.

Changes made in the revised manuscript

- **Results**: reorganized the original short numbered sections into broader thematic subsections covering (i) Esc2/Rad57 chromatin association, (ii) Esc2/Rad57-defined gene classes and RNA changes, (iii) Pol II accumulation, (iv) Sit4/Rrd1 association, (v) mitochondrial defects and growth phenotypes, and (vi) acute Rad57 depletion by anchor-away.
- **Figure organization**: revised the arrangement of main and supplementary figures to match the new Results structure.

Other concern 9

Reviewer comment:

For data analysis, the number of replicates should be clearly indicated and the way how replicates were taken into account in analysis and the comparison between the replicates should be systematically presented.

Response:

We agree. In the revised manuscript, we have clarified the number of biological replicates used in each experiment and have revised the Methods and figure legends to describe more explicitly how replicates were incorporated into the analyses. We have also improved the presentation of replicate consistency, including additional QC-style analyses where relevant.

Changes made in the revised manuscript

- **Methods**: clarified the number of biological replicates and how they were used in ChIP-seq, RNA-seq, ChIP-qPCR, RT-qPCR, and growth analyses.
- **Figure legends**: revised to indicate replicate numbers and the way summary statistics are displayed.
- **Supplementary figures**: added or revised PCA plots and Pearson correlation heatmaps for ChIP-seq, RNA-seq, Pol II ChIP-seq, and anchor-away RNA-seq datasets to document replicate consistency.

Other concern 10

Reviewer comment:

Introduction, p. 3 - – recent paper on promoter-proximal pausing across evolution should be cited (Chivu NSMB 2026) and the text of introduction should be modified accordingly.

Response:

We thank the reviewer for this suggestion. We have added the suggested citation and revised the Introduction accordingly to better place our work in the broader context of Pol II regulation across species.

Changes made in the revised manuscript

- **Introduction**, first paragraph: added the citation to **Chivu et al. (2026)**. (**p. 3, line 11**)
- **Introduction**, opening section on Pol II regulation: revised the text to place budding-yeast gene-body control in the broader evolutionary context of promoter-proximal pausing across eukaryotes. (**p. 3, lines 3-11**)

Other concern 11

Reviewer comment:

Figure S5C - –o comment on the scale of the graph, the signal is not flat.

Response:

We agree and have clarified both the figure and legend for **Figure S5C**. The panel now explicitly indicates the signal scale used in the heatmap, and we clarify that the displayed pattern reflects low but non-flat **Ptc2** ChIP-seq signal across the analyzed regions.

Changes made in the revised manuscript

- Figure S5C: added an explicit labeled signal scale to the heatmap.
- Figure S5 legend: clarified that Ptc2 shows a low but non-flat signal pattern and does not display a comparably clear gene-body enrichment pattern.

Other concern 12

Reviewer comment:

The scale in Fig S5C should be indicated.

Response:

We agree. A labeled signal scale has now been added directly to **Figure S5C**.

Changes made in the revised manuscript

- **Figure S5C**: added a labeled heatmap scale bar indicating the signal range used for visualization.
- **Figure S5 legend**: added a description of how color intensity corresponds to CPM-normalized ChIP-seq signal.

Other concern 13

Reviewer comment:

There is a problem with the text font on page 21 (additional information, author contribution), page 26 (Fig. 1 legend), page 29 (Figure S1 legend).

Response:

We thank the reviewer for noticing this. These formatting issues have been corrected in the revised manuscript.

Changes made in the revised manuscript

- **Additional information / author contributions**
- **Figure 1 legend**
- **Figure S1 legend**

  All font inconsistencies identified by the reviewer were corrected in the revised manuscript.

Other concern 14

Reviewer comment:

To correct "g“ne-wise" ”o "g“nome-wide" ”n Table S2, 3, 4, 5 titles.

Response:

We agree and have corrected these titles in the revised manuscript.

Changes made in the revised manuscript

- **Table tiles** : corrected **“gene-wise”** or **“genome-wide.”**

Other concern 15

Reviewer comment:

Figure S1D - –roblem with headings, Figure S2E - –isplay problem.

Response:

We thank the reviewer for pointing this out. These display and heading problems have been corrected in the revised figures.

Changes made in the revised manuscript

- **Figure S1**: corrected panel heading/layout issues.
- **Figure S2E**: corrected the display problem in the revised figure.

Reviewer #2

We thank the reviewer for the constructive and insightful comments. We appreciate the reviewer's emphasis on improving the directness of the analyses and clarifying the relationship between Esc2/Rad57 occupancy, Pol II accumulation, and transcriptional output. In the revised manuscript, we substantially strengthened this aspect of the study by adding direct genome-wide correlation analyses, expression-stratified metagene analyses, locus-specific validation by Pol II ChIP-qPCR and RT-qPCR, and a revised interpretation of the growth phenotype. We also improved the presentation and QC of the RNA-seq datasets. As detailed below, we believe these revisions directly address the reviewer's major concerns.

Major point 1

Reviewer comment:

The authors categorize genes into groups (C1-C3) based on Esc2/Rad57 signal intensity... Rather than relying primarily on gene clustering approaches, the authors should provide a direct gene-by-gene correlation... Specifically: (a) genome-wide correlation analysis between Esc2/Rad57 chromatin occupancy and mRNA levels; (b) metagene profiles of Esc2/Rad57 binding across gene cohorts categorized by expression level.

Response:

We agree with this point and have revised the manuscript accordingly. In the revised manuscript, we added direct gene-by-gene comparisons between Esc2/Rad57 chromatin occupancy and steady-state RNA abundance across all genes, as well as metagene analysis of Esc2/Rad57 binding over a high-expression gene cohort. These analyses showed that Esc2/Rad57 occupancy exhibits only a weak gene-by-gene correlation with steady-state RNA abundance, while metagene analysis demonstrated clear enrichment of Esc2/Rad57 over highly expressed genes. We have

therefore revised the interpretation accordingly: rather than arguing for a simple monotonic relationship between Esc2/Rad57 binding and transcript output, we now conclude more conservatively that Esc2/Rad57 are preferentially associated with highly active gene bodies, whereas the transcriptional consequences of their loss are selective and not uniformly reflected by steady-state RNA levels.

Changes made in the revised manuscript

- **Figure S1C** and corresponding legend: added direct gene-by-gene comparisons between Esc2/Rad57 gene-body ChIP signal and steady-state RNA abundance.
- **Figure 1B** and corresponding legend: added metagene analysis over a high-expression gene cohort.
- **Results**, subsection describing Esc2/Rad57 chromatin association and RNA consequences: revised the interpretation to state that Esc2/Rad57 show weak genome-wide gene-by-gene correlation with RNA abundance despite preferential enrichment over highly expressed genes. (**p. 6, lines 18-23**)

Major point 2

Reviewer comment:

There are significant concerns regarding the quality and presentation of the RNA-seq data (Figure S2)... the single mutant plots appear to be missing the vast majority of genes... The PCA analysis shows high variance between the WT replicates and between the esc2 single mutant replicates.

Response:

We agree that the previous presentation of the RNA-seq data was not sufficiently clear. In the revised manuscript, we clarified the RNA-seq analysis workflow, thresholds, and filtering criteria in the Methods and figure legends, and we revised the presentation so that the overall distribution of RNA-seq changes is more clearly visible. These revisions make it clearer that the sparse appearance of some single-mutant datasets reflects the relatively limited effect sizes and higher variability of those datasets, rather than the absence of most genes from the analysis. At the same time, we revised the text to interpret the single-mutant RNA-seq results more cautiously and to place greater emphasis on the more robust and reproducible phenotypes observed in the *esc2*Δ *rad57*Δ double mutant.

Changes made in the revised manuscript

- **Methods**, RNA-seq subsection: clarified the analysis workflow, thresholds, and filtering criteria. (**p. 19, lines 13-20**)
- **Figure S2A–E** and corresponding legends: revised the presentation of RNA-seq quality

control and differential expression analyses.

- **Results**, subsection describing RNA-seq changes: added the statement that single-mutant datasets show limited transcriptome-wide effects and greater inter-replicate variability than the *esc2*Δ *rad57*Δ double mutant. (**p.7, lines 12-21**)

Major point 3

Reviewer comment:

In Figure S2, the authors claim that upregulated genes in the esc2Δ rad57Δ mutant are enriched in Clusters 1 and 2. However, the actual overlap appears to be very limited... The authors should clarify why the vast majority of genes bound by these factors show no detectable transcriptional change upon deletion.

Response:

We agree that the binary overlap between Esc2/Rad57-bound genes and significantly upregulated genes in *esc2*Δ *rad57*Δ cells is limited, and that our original wording overstated the implication of this overlap. In the revised manuscript, we have therefore clarified that enrichment of upregulated genes in Clusters 1 and 2 should not be interpreted as meaning that most Esc2/Rad57-bound genes undergo detectable RNA increases upon deletion. Rather, we interpret Esc2/Rad57 occupancy as marking highly active gene-body chromatin contexts, whereas the transcriptional consequences of Esc2/Rad57 loss are selective and become evident only for a subset of these genes. Consistent with this revised interpretation, we now place less emphasis on binary overlap counts alone and instead incorporate direct gene-by-gene comparative analyses and cluster-level comparisons relating Esc2/Rad57 occupancy, Pol II behavior, and RNA abundance.

Changes made in the revised manuscript

- **Results, subsection describing Esc2/Rad57-defined gene classes and RNA-seq changes:** revised the text to state explicitly that most Esc2/Rad57-bound genes do not show overt RNA increases upon deletion. (**p. 7, line 22-p. 8, line 7**)
- **Figure 2C legend:** revised to present the RNA-seq changes across ER-C1–ER-C3 gene classes as supportive of selective transcriptional consequences, rather than as evidence for a uniform response of all Esc2/Rad57-bound genes.
- **Results, subsection describing Pol II accumulation:** added cluster-level, overlap-based, and continuous-variable analyses linking Esc2/Rad57 occupancy to Pol II accumulation and selective RNA consequences (**p. 9, lines 7–13**).

Major point 4

Reviewer comment:

The authors report that acute Rad57 depletion using an anchor-away system... does not recapitulate the transcriptome alterations observed in the stable double-deletion mutant. However, the variance between biological replicates in Figure S4C appears to be very high... The authors should provide a Pearson correlation matrix for all RNA-seq samples... and clarify why the volcano plots display such a limited number of genes.

Response:

We agree that the original presentation of the anchor-away RNA-seq dataset was not sufficiently transparent. In the revised manuscript, we have added replicate-consistency analyses, including both PCA and a Pearson correlation matrix for all RNA-seq samples, to make sample-to-sample relationships more explicit. These analyses show that the anchor-away dataset contains appreciable replicate variability, including one −Rap sample with markedly reduced correlation to the other replicates. We have therefore interpreted this dataset more cautiously in the revised manuscript. We also clarified the workflow used for differential expression analysis and revised the presentation of the volcano plot. The limited number of significant genes under acute depletion conditions is now presented as reflecting both the relatively modest transcriptome effect of short-term Rad57 depletion under the tested conditions and the reduced statistical power associated with replicate variability. Importantly, we no longer interpret the weak overlap between the anchor-away dataset and the *esc2*Δ *rad57*Δ double-mutant transcriptome as a mechanistic contradiction. Instead, we now conclude more conservatively that acute Rad57 nuclear depletion in the esc2Δ background does not fully phenocopy the chronic double-mutant state under the tested conditions, likely reflecting the difference between acute perturbation and the cumulative consequences of stable loss.

Changes made in the revised manuscript

- **Figure S7C–E** and corresponding legends: added PCA, Pearson correlation heatmap, and revised volcano plot presentation for the anchor-away RNA-seq dataset.
- **Results**, subsection beginning *"Acute nuclear depletion of Rad57 causes only limited transcriptome changes..."*: revised the text to emphasize limited transcriptome-wide effects and appreciable replicate variability. **(p. 12, line 14-p. 13, line 5)**
- **Discussion**, paragraph discussing acute versus chronic perturbation: revised to state that acute Rad57 depletion does not fully phenocopy the cumulative consequences of stable loss. (**p. 15, lines 10-14**)
- **Methods**, RNA-seq subsection: clarified the differential expression workflow for the anchor-away dataset. (**p. 17, lines 10-14**)

Major point 5

Reviewer comment:

In Figure 2, to clarify the relationship between these recombination factors and Pol II dynamics, the authors should perform a direct gene-by-gene correlation between Esc2/Rad57 recruitment levels (ChIP-seq signal) and Pol II occupancy in the esc2Δ rad57Δ mutant.

Response:

We agree, and this point has now been addressed in the revised manuscript. We added direct gene-by-gene comparative analyses relating Esc2/Rad57 occupancy to Pol II accumulation in esc2Δ rad57Δ cells. These analyses revealed only a weak positive genome-wide correlation, indicating that the relationship between Esc2/Rad57 localization and Pol II accumulation is not captured by a simple gene-by-gene proportional model. We therefore interpret this relationship more conservatively in the revised manuscript, based on the combination of direct comparative analyses, cluster-level patterns, overlap analyses, and locus-specific validation, rather than on binary overlap alone.

Changes made in the revised manuscript

- Figure S3D and corresponding legend: added direct gene-by-gene comparisons between Esc2/Rad57 gene-body ChIP-seq signal and Pol II accumulation in *esc2*Δ *rad57*Δ cells.
- Results, subsection beginning "Loss of Esc2/Rad57 increases Pol II gene-body occupancy...": revised to state that the relationship between Esc2/Rad57 localization and Pol II accumulation is weakly positive but not globally proportional. **(p. 9, lines 8-11)**

Major point 6

Reviewer comment:

The authors fail to establish a causal link between the growth defects shown in Figure 4 and the reported transcriptional changes... As presented, the growth phenotype could reflect secondary consequences of genomic instability, recombination defects, or broader cellular stress rather than a direct transcriptional effect of Esc2 and Rad57 loss.

Response:

We agree. In the revised manuscript, we have substantially moderated the interpretation of the growth phenotype. We now explicitly state that the slow-growth phenotype likely reflects multiple contributing factors, including mitochondrial dysfunction and broader genome-maintenance defects, and we no longer present it as a direct consequence of altered transcription alone. At the same time, we distinguish these broader physiological consequences from the molecular phenotypes characterized in this study, such as abnormal Pol II accumulation and altered

transcriptional homeostasis. The corresponding figure/model and Discussion have been revised accordingly.

Changes made in the revised manuscript

- **Results**, subsections beginning *"Reduced mitochondrial transcripts in esc2Δ rad57Δ are associated with mitochondrial DNA loss"* and *"Chronic loss of Esc2/Rad57 is associated with slow growth"*: clarified that mitochondrial dysfunction and slow growth are broader physiological consequences and not direct readouts of altered Pol II distribution. (**p. 11, line 13-p. 12, line 2; p. 12, lines 4-12**)
- **Discussion**, paragraph beginning *"Finally, esc2Δ rad57Δ cells exhibit mitochondrial DNA loss..."*: revised to state explicitly that the slow-growth phenotype is likely multifactorial. (**p. 15, lines 15-2**2)
- **Figure 5B** and corresponding legend: revised the model to avoid presenting growth defects as a direct linear consequence of altered transcription alone.

Minor point 1

Reviewer comment:

The manuscript lacks a clear rationale for specifically investigating the association of Esc2 and Rad57 with chromatin.

Response:

We agree that the rationale was not stated clearly enough in the previous version. We have revised the Introduction to more explicitly explain the observations and prior work that motivated us to examine Esc2 and Rad57 chromatin association in the present study.

Changes made in the revised manuscript

- **Introduction**, paragraph describing Esc2 and Rad57: added the rationale linking prior work on GAGsi-mediated transcriptional silencing (Masumoto *et al*, 2024) to the present analysis of Esc2/Rad57 chromatin association. (**p. 4, lines 14-18**)

Minor point 2

Reviewer comment:

The authors should briefly explain the mechanism of the anchor-away system and clarify the rationale for using rapamycin to induce nuclear depletion.

Response:

We agree and have added a brief explanation of the anchor-away system and the rationale for rapamycin treatment in the revised manuscript.

Changes made in the revised manuscript

- **Results**, subsection beginning *"Acute nuclear depletion of Rad57 causes only limited transcriptome changes..."*: added a brief explanation of the anchor-away strategy and the use of rapamycin to induce nuclear depletion. (**p. 12, lines 16-20**)
- **Figure S7A legend**: clarified the principle of the **Rad57-FRB anchor-away system** and rapamycin-dependent relocalization.

Reviewer #3

We thank the reviewer for the thoughtful and constructive comments. We appreciate the reviewer's emphasis on the physiological interpretation of Esc2/Rad57 enrichment in ORFs, the need to clarify the meaning of increased Pol II occupancy in esc2Δ rad57Δ cells, and the importance of additional locus-specific validation. In the revised manuscript, we addressed these concerns by moderating our mechanistic claims, adding Pol II ChIP-qPCR and RT-qPCR validation at representative loci, incorporating more direct comparative analyses, and clarifying the interpretive scope of the Sit4/Rrd1 model. As detailed below, we believe these revisions substantially strengthen the manuscript.

General concern 1

Reviewer comment:

Although the authors' finding that recombination-related factors are enriched in coding regions is interesting, several issues remain unresolved. A key unresolved question is whether the enrichment of Esc2 and Rad57 in ORFs, along with increased Pol II occupancy in coding regions in the double mutant, has physiological significance. Although the authors report growth defects in the double mutant, these may result from disruptions in other functions.

Response:

We agree that the original manuscript overstated the physiological interpretation of the growth phenotype relative to the transcriptional data. In the revised manuscript, we have moderated this interpretation throughout the Results, Discussion, and model figure. We no longer present slow growth as a direct consequence of altered Pol II distribution alone. Instead, we now explicitly acknowledge that mitochondrial dysfunction and broader genome-maintenance defects likely contribute to the physiological phenotype of *esc2Δ rad57Δ* cells. At the same time, we interpret the ORF enrichment of Esc2 and Rad57 more specifically as a molecular feature associated with Pol II-rich, highly active gene bodies and with the maintenance of transcriptional homeostasis, rather than as a direct explanation for all physiological defects observed in the double mutant.

Changes made in the revised manuscript

- **Results**, subsections describing mitochondrial DNA loss and slow growth: revised to distinguish molecular phenotypes from broader physiological consequences. (**p. 12, lines 5-12**)
- **Discussion**, paragraph beginning *"Finally, esc2Δ rad57Δ cells exhibit mitochondrial DNA loss..."*: clarified that the physiological phenotype is likely multifactorial and not directly explained by altered Pol II distribution alone. (**p. 15, lines 15-22**)
- **Discussion**, opening paragraph: revised to interpret Esc2/Rad57 ORF enrichment primarily as a molecular signature associated with Pol II-rich, highly active gene bodies and transcriptional homeostasis. (**p. 14, lines 2-10**)
- **Figure 5B** and corresponding legend: revised the working model to avoid presenting physiological defects as a direct linear consequence of altered Pol II distribution.

General concern 2

Reviewer comment:

The second unresolved issue concerns what the increase in Pol II levels indicates regarding transcription activity... higher Pol II levels in ORFs correlate with higher transcriptional activity... However, this is not reflected in RNA-seq data... Alternatively, it could suggest stalled or slow-elongating Pol II...

Response:

We agree that increased Pol II occupancy in gene bodies does not, by itself, uniquely define the functional state of the polymerase. In the revised manuscript, we therefore take a more cautious interpretive approach. Rather than claiming that increased Pol II occupancy occurs "without changes in transcriptional activity" in a strict sense, we now state more specifically that increased Pol II occupancy does not uniformly translate into increased steady-state RNA levels. To support this point more directly, we added locus-specific validation by Pol II ChIP-qPCR and RT-qPCR at representative genes, which show that the relationship between Pol II accumulation and RNA output is gene-dependent. We also revised the Discussion to explicitly acknowledge that our data do not distinguish among all possible mechanistic states of the accumulated Pol II, including the possibilities of slow-moving, stalled, or otherwise non-productively retained polymerase. Accordingly, the current study is framed as defining a chromatin-level phenomenon and its transcript-level consequences, rather than assigning a definitive biochemical state to the accumulated Pol II.

Changes made in the revised manuscript

- **Results**, subsection beginning *"Loss of Esc2/Rad57 increases Pol II gene-body occupancy..."*: added locus-specific Pol II ChIP-qPCR and RT-qPCR analyses showing that increased Pol II occupancy is not uniformly accompanied by proportional RNA changes. (**p. 9, lines 4-8**)
- **Figure 3F–H** and corresponding legends: revised to emphasize the gene-dependent relationship between Pol II accumulation and steady-state RNA output.
- **Discussion**, paragraph beginning *"The increased Pol II occupancy observed..."*: revised to state explicitly that the biochemical state of the accumulated Pol II remains unresolved and may include slow-moving, stalled, or otherwise non-productively retained polymerase. (**p. 15, lines 4-8**)

Comment on Sit4/Rrd1 genome-wide profiling

Reviewer comment:

Because this paper does not establish a clear role for Esc2 and Rad57 enrichment in ORFs, conducting genome-wide ChIP-seq for Sit4 and Rdr1 could help in implicating their function in regulating Sit4 and Rdr1 occupancies.

Response:

We appreciate this suggestion. In fact, genome-wide ChIP-seq analyses for Sit4 and Rrd1 are already included in the revised manuscript. However, we agree that the functional interpretation of these datasets needed to be clarified. In the revised version, we have therefore revised the text to better explain how the Sit4/Rrd1 occupancy patterns relate to Esc2/Rad57-associated, Pol II-rich gene clusters, while keeping the mechanistic model appropriately cautious and provisional. At the same time, in accordance with the editor's decision letter, we prioritized strengthening the direct relationship among Esc2/Rad57 localization, Pol II accumulation, and transcript output through additional comparative analyses and locus-specific validation.

Changes made in the revised manuscript

- **Figure 4A–E** and corresponding legends: clarified the genome-wide relationship between **Sit4/Rrd1** occupancy, **Esc2/Rad57** association, and Pol II-rich gene clusters.
- **Results**, subsection beginning *"Sit4 and Rrd1 are associated with Esc2/Rad57-enriched gene bodies..."*: revised to explain that **Rrd1** shows a weaker and less sharply gene-body-centered pattern than Esc2, Rad57, and Sit4, while remaining associated with the same chromatin context. (**p. 9, line 20-p. 10, line 23**)
- **Discussion**, paragraph beginning *"Our genome-wide and locus-specific analyses further connect..."*: revised to present the Sit4/Rrd1 model as provisional and mechanistically cautious. (**p. 14, line 19-p. 15, line 3**)

Additional comment 1

Reviewer comment:

Authors should also show protein levels for the recombination-related factors to ensure that the low enrichment of Rad51 and Rad55 is not due to their lower expression relative to Rad57 and Esc2.

Response:

We agree that this was an important control. In the revised manuscript, we added anti-Flag immunoblotting for the C-terminally 4Flag-tagged Esc2, Rad57, Rad51, and Rad55 strains used in the ChIP analyses. These blots confirm that all four tagged proteins are detectable. We note, however, that Esc2-4Flag was observed predominantly as slower-migrating species and that Rad51-4Flag migrated faster than expected. We therefore use these blots primarily as evidence of detectability rather than as a strict quantitative comparison of protein abundance. Accordingly, in the revised text we no longer interpret the weaker Rad51/Rad55 ChIP signals simply as a consequence of absent or undetectable protein, while also avoiding a strict quantitative comparison of ChIP signal based solely on immunoblot intensity.

Changes made in the revised manuscript

- **Figure S1A** and corresponding legend: added anti-Flag immunoblotting for **Esc2-4Flag, Rad57-4Flag, Rad51-4Flag, and Rad55-4Flag**.
- **Results**, subsection beginning *"Esc2 and Rad57 preferentially associate..."*: added the statement that all four tagged proteins were detectable, while noting the atypical migration patterns of Esc2-4Flag and Rad51-4Flag. (**p. 6, lines 8-11**)
- **Discussion**: revised to avoid interpreting weaker Rad51/Rad55 ChIP signals simply as a consequence of absent or undetectable protein. (**p. 14, lines 11-18**)

Additional comment 2

Reviewer comment:

ORFs of several highly expressed genes are classified as hyper-ChIP-able regions. The authors identify 41 genes that overlap between Esc2-Rad57 and Pol II cluster 1 (Fig. 2C). Do the authors observe statistically significant enrichment when the hyper-ChIP-able regions are removed?

Response:

We agree that this is an important concern. In the analyses underlying Fig. 3D (formerly referred to as Fig. 2C in the previous version), the ChIP-seq signal was already evaluated using IP-minus-no-tag IP values, such that background signal was subtracted genome-wide rather than only for the overlapping genes. In addition, in the revised manuscript we now include explicit no-tag/noIP

control analyses and acknowledge that highly transcribed loci can still exhibit elevated background-like behavior in ChIP-based assays. We therefore interpret the Esc2/Rad57 enrichment more conservatively as signal above background at highly transcribed genes, rather than as evidence entirely free from potential hyper-ChIP-related contribution.

Changes made in the revised manuscript

- **Figure 1A** and corresponding legend: added representative browser views showing **Esc2-4Flag** and **Rad57-4Flag** together with no-tag/noIP controls at highly transcribed loci.
- **Figure 1B** and corresponding legend: added no-tag control profiles to metagene analyses.
- **Results**, subsection beginning *"Esc2 and Rad57 preferentially associate..."*: revised to state explicitly that no-tag/noIP controls show modest signal at ChIP-prone loci, whereas Esc2/Rad57 signals reproducibly exceed this background. (**p. 6, lines 4-8**)

Additional comment 3

Reviewer comment:

The authors should show Pol II occupancy by ChIP-qPCR for at least a couple of genes that reveal high Pol II ORF occupancy in the double mutant, and also RNA levels for those genes by qRT-PCR.

Response:

We agree, and this has now been addressed directly. In the revised manuscript, we added Pol II ChIP-qPCR at representative highly transcribed loci together with RT-qPCR analysis of the corresponding transcripts. These locus-specific analyses confirm increased Pol II occupancy in **esc2Δ rad57Δ** cells while also showing that the effect on steady-state RNA output is gene-dependent rather than uniform. This addition directly addresses the reviewer's request and strengthens the connection between the genome-wide analyses and the locus-specific behavior of representative genes.

Changes made in the revised manuscript

- **Figure 3F and 3G** and corresponding legends: added RT-qPCR and Pol II ChIP-qPCR analyses at representative highly transcribed loci.
- **Results**, subsection beginning *"Loss of Esc2/Rad57 increases Pol II gene-body occupancy..."*: added locus-specific validation showing increased Pol II occupancy together with gene-dependent RNA outcomes. (**p. 9, lines 4-7**)
- **Methods**, subsections ChIP-qPCR and RNA RT-qPCR: added experimental details for the new validation assays, with primer information provided in Table S6. (**p. 18, line 18-p. 19, line 1; p. 19, line 22-p. 20 line 1**)

Additional comment 4

Reviewer comment:

The authors find that Esc2, Rad57, and Sit4 are enriched in ORFs and present the enrichment as heatmaps (Fig. 3A). Are the genes sorted in the same order, or are they sorted by their individual enrichment levels? It would be important to show a correlation plot for Pol II and the three factors' ORF occupancies.

Response:

We agree that this required clarification. In the revised manuscript, we now explicitly state in the figure legend and Methods how the genes were ordered in the heatmaps. Specifically, the gene clusters defined in Figure 4C were used as the common framework for subsequent analyses. We then directly examined Pol II accumulation in *esc2*Δ *rad57*Δ cells across these same clusters in Figure 4D and 4E, providing a quantitative comparison between factor-associated gene groups and Pol II accumulation. These additions reduce the ambiguity of the original presentation and clarify the relationship between Esc2/Rad57/Sit4/Rrd1-enriched gene clusters and abnormal Pol II accumulation in the mutant background.

Changes made in the revised manuscript

- **Figure 4 legend**: added an explicit statement that genes were displayed in a common order across the heatmap panels based on the clustering shown in **Figure 4C**.
- **Methods**, ChIP-seq / clustering description: clarified that gene clusters defined from the **Esc2/Rad57/Sit4/Rrd1** occupancy patterns were used as the common ordering framework for downstream visualization and comparison with Pol II accumulation. (**p. 19, lines 3-10**)
- **Figure 4D and 4E** and corresponding legends: added the quantitative comparison linking the same cluster framework to Pol II accumulation in ***esc2*Δ *rad57*Δ** cells.

Additional comment 5

Reviewer comment:

Fig. 3C shows heatmaps that reveal 39 genes enriched across all four factors. Is this significant?

Response:

We agree that the significance of this group was not sufficiently clear in the original presentation. In the revised manuscript, we no longer rely on the visual overlap alone. Instead, the **39-gene group (ERSR-C2)** is evaluated quantitatively in **Figure 4E**, which shows that this cluster displays significantly elevated Pol II accumulation in **esc2Δ rad57Δ** cells compared with the background cluster. We revised the text accordingly so that this group is interpreted on the basis

of its statistically supported Pol II behavior rather than on visual overlap alone.

Changes made in the revised manuscript

- **Results**, subsection beginning *"Sit4 and Rrd1 are associated with Esc2/Rad57-enriched gene bodies..."*: revised to state that the highly co-associated gene class is interpreted on the basis of its quantitative Pol II behavior rather than visual overlap alone. (**p. 10, lines 16-23**)
- **Figure 4E** and corresponding legend: emphasized the statistically supported increase in Pol II accumulation in the strongest four-factor cluster.

Additional comment 6

Reviewer comment:

Fig 3F-G. The authors should show Pol II levels at these genes.

Response:

We agree, and we have strengthened the connection between these loci and Pol II in the revised manuscript. Specifically, we now show Pol II occupancy directly at a representative Esc2/Rad57-associated locus (*TDH3*) using a genome-browser view (Figure 3A), and we further support this locus-level relationship by Pol II ChIP-qPCR at representative highly transcribed genes, including *CDC19* and *TPI1* (Figure 3G). These additions make the link between Esc2/Rad57-associated loci and Pol II accumulation more direct in the revised figure set.

Changes made in the revised manuscript

- **Figure 3A** and corresponding legend: added representative genome-browser views showing increased Pol II occupancy at the *TDH3 locus in esc2Δ rad57Δ cells*.
- **Figure 3G** and corresponding legend: added Pol II ChIP-qPCR at representative highly transcribed genes, including *CDC19* and *TPI1*.
- **Results**, subsection beginning *"Loss of Esc2/Rad57 increases Pol II gene-body occupancy..."*: revised to connect the representative loci more directly to Pol II accumulation. (**p. 8, lines 10-13; p. 9, lines 4-7**)

Additional comment 7

Reviewer comment:

The authors propose that Rad57/Esc2 prevents hyperaccumulation of Pol II. If that is the case, one would observe a significant increase in mRNA and Pol II levels at the Rad57/Esc2-enriched genes in the double mutant.

Response:

We appreciate this point and agree that our previous wording did not sufficiently distinguish between Pol II occupancy and transcript output. In the revised manuscript, we now explicitly state that increased Pol II occupancy does not uniformly correspond to increased steady-state RNA levels. The expectation that higher Pol II levels should necessarily lead to higher mRNA output would hold only if the accumulated polymerase were uniformly engaged in productive transcription. Our data do not support such a simple one-to-one relationship. Instead, we interpret the results more cautiously as indicating that Esc2/Rad57 help restrain excess Pol II accumulation at highly transcribed genes, whereas the effect on steady-state RNA accumulation is selective and locus-dependent. The added locus-specific Pol II ChIP-qPCR and RT-qPCR analyses directly support this revised interpretation.

Changes made in the revised manuscript

- **Results**, subsection beginning *"Loss of Esc2/Rad57 increases Pol II gene-body occupancy..."*: revised to state explicitly that increased Pol II occupancy is not uniformly accompanied by proportional increases in steady-state RNA output. (**p. 9, lines 4-8**)
- **Figure 3F–H** and corresponding legends: added locus-specific RT-qPCR and Pol II ChIP-qPCR analyses together with the genome-wide comparison between Pol II occupancy and RNA change.
- **Discussion**, paragraph beginning *"The increased Pol II occupancy observed..."*: revised to state that elevated Pol II occupancy should not be interpreted as a simple proxy for uniformly increased productive transcription, but rather as abnormal polymerase accumulation with selective transcript-level consequences. (**p. 15, line 4-14**)

Additional comment 8

Reviewer comment:

The authors use C1, C2, and C3 clusters to define Pol II occupancy (Fig 2B) and same nomenclature for the occupancies of Esc2 and Rad57. It would be easier for the reader if they named clusters as Pol II-C1 cluster or Esc2-C1 cluster.

Response:

We agree and have revised the nomenclature throughout the revised manuscript and figures to distinguish more clearly between **Pol II-defined** and **Esc2/Rad57-defined** clusters.

Changes made in the revised manuscript

- **Main text** and **figure legends**: revised the nomenclature to distinguish Esc2/Rad57-defined clusters (ER-C1 to ER-C3) from Pol II-defined clusters (Pol II-C1 to Pol II-C3).

- **Figure 2** and corresponding legend: updated cluster names to ER-C1 to ER-C3.
- **Figure 3** and corresponding legend: updated cluster names to Pol II-C1 to Pol II-C3.
- **Figure 4** and corresponding legend: clarified the distinct nomenclature for Esc2/Rad57/Sit4/Rrd1-defined clusters (ERSR-C2, ERSR-C1, and ERSR-C0).

July 7, 2026

Re: Life Science Alliance manuscript #LSA-2026-03673-TR

Dr. Hiroshi Masumoto
Nagasaki University
Biomedical Research Support Center (BRSC)
1-12-4 Sakamoto
Sakamoto
Nagasaki, Nagasaki 8528523
Japan

Dear Dr. Masumoto,

Thank you for submitting your revised manuscript entitled "Esc2 and Rad57 contribute to RNA polymerase II distribution at highly transcribed genes" to Life Science Alliance.

The manuscript has been seen by two of the original reviewers whose comments are appended below. While the reviewers have noted your revisions, they have also highlighted several pending yet pertinent issues.

-Both reviewers have commented that while a direct role for Esc2/Rad57 in regulating Pol II distribution during transcription is claimed in the ms text, no supporting evidence is presented here. We agree that this claim must be moderated throughout the text. We also agree with both reviewers that you must revisit your presented model and limit conclusions to only those based on evidence (Reviewer 1, point 6 and Reviewer 2, last sentence).
-Reviewer 1 also raises an important technical point on the new data added in your revisions (points 2 and 3) that we agree you must include controls for figure 1C and 4A, and negative control region for ChIP-qPCR. Further you must address the point of this reviewer on comparison of regions between ChIP and RT-CPR (point 3).
-We also concur with Reviewer 1 that you must provide clarifications in the ms text for point 7 raised by the reviewer.
-Finally, we understand (from page 4 of your response letter) that your approach did not use exogenous spike-material, and therefore you cannot implement normalisation of the ChIPseq data. We recommend that you clarify in the next text and include this point from the reviewer as a potential limitation of your work.

Our general policy is that papers are considered through only one revision cycle; however we are open to one additional short round of revision. Please submit the final revision (with changes highlighted) within one month, along with a letter that includes a point by point response to the reviewer comments.

To upload the revised version of your manuscript, please log in to your account: https://lsa.msubmit.net/cgi-bin/main.plex
You will be guided to complete the submission of your revised manuscript and to fill in all necessary information.

Please pay attention to adhere to our editorial requirements for revisions:

A. THESE ITEMS ARE REQUIRED FOR REVISIONS

-- A letter addressing the reviewers' comments point by point.

-- An editable version of the final text (.DOC or .DOCX) is needed for copyediting (no PDFs).

-- High-resolution figure, supplementary figure and video files uploaded as individual files: See our detailed guidelines for preparing your production-ready images, https://www.life-science-alliance.org/authors

-- Summary blurb (enter in submission system): A short text summarizing in a single sentence the study (max. 200 characters including spaces). This text is used in conjunction with the titles of papers, hence should be informative and complementary to the title and running title. It should describe the context and significance of the findings for a general readership; it should be written in the present tense and refer to the work in the third person. Author names should not be mentioned.

B. MANUSCRIPT ORGANIZATION AND FORMATTING:

Full guidelines are available on our Instructions for Authors page, https://www.life-science-alliance.org/authors

Thank you for this interesting contribution to Life Science Alliance.

Sincerely,

Sarita Hebbar, PhD
Scientific Editor
Life Science Alliance
http://www.lsajournal.org

Reviewer #1 (Comments to the Authors (Required)):

In this revised version of the manuscript, the authors tried to respond to criticism from the reviewers by modifying the text and by including some additional results, focusing on the main points highlighted by the editor. However, the responses are limited and they do not reinforce the conclusions and do not allow to propose potential mechanisms.

Specific points:
1. The major concerns remain unanswered on potential role of Esc2 and Rad57 in Pol II dynamics and transcriptional activity, and a potential link with growth phenotypes of deletion strains.

2. Even though the authors added no tag IP control in Fig 1A, B, this control is missing in other figures (for example Fig 1C, 4A etc) and is not included systematically in their analyses. Moreover, differences between ChIP signals for homologous-recombination components that the authors consider as enriched on highly transcribed genes and those that they consider as non-enriched are unclear. It is unclear how the authors conclude that Rrd1 is enriched, but Rad51 and Rad55 or Ptc2 are non-enriched. A rigorous comparison with control IP condition is missing.

3. ChIP-qPCR and RT-qPCR results added on revision step (Fig 3F, G, 4F, G) were obtained for very few regions, only two regions that are not even the same between ChIP and RT-PCR on Fig 3F, G. The variability for Pol II ChIP-qPCR ratio on CDC19 (Fig 3G) is very high and incompatible with any possible conclusion and publication standard. No negative control region for ChIP-qPCR experiments was included that is needed to conclude on enrichment.

4. Since no appropriated normalization of ChIPseq data was done, different samples cannot be compared in a correct way to conclude about changes between strains. Moreover, Fig 3B for Pol II in double deletion mutant is problematic with very different baselines for three cluster metagene profiles.

5. The authors provided results on mitochondrial problems in esc2Δ rad57Δ that further reinforces the potential indirect effects unrelated to ChIPseq results.

6. The model figure contains many points without any evidence provided, for example for Pol II release by Rrd1 or reduced transcription (no direct measurements were done) or link with growth phenotypes.

7. Supplementary figure panels are not always cited and the quality of some figures should be improved, for example Fig S2D. Replicate analysis reveals several problems, for example low correlation between WT r1 and r2 (0.55) even lower than between WT r1 and esc2 rad57 replicates (0.66, 0.62). The AA rad57 experience is difficult to interpret since there is a problem with -Rap replicate 2.

Reviewer #2 (Comments to the Authors (Required)):

The authors have addressed the major points raised in my initial review. Specifically, they performed the requested genome-wide and gene-by-gene correlation analyses to directly test the relationship between Esc2/Rad57 occupancy and transcriptional output. As these new analyses revealed that the direct correlations are relatively weak or highly restricted, the authors have revised the manuscript. However, the authors still imply a direct role for Esc2/Rad57 in regulating Pol II distribution during transcription (e.g., in the abstract). Because the data remains correlative, an active or mechanistic role for Esc2 and Rad57 in regulating Pol II dynamics is not fully supported. I recommend that the authors perform texts revision to soften these specific claims and their model.

Rebuttal

Dear Prof. Sarita Hebbar and Reviewers,

-Both reviewers have commented that while a direct role for Esc2/Rad57 in regulating Pol II distribution during transcription is claimed in the ms text, no supporting evidence is presented here. We agree that this claim must be moderated throughout the text. We also agree with both reviewers that you must revisit your presented model and limit conclusions to only those based on evidence (Reviewer 1, point 6 and Reviewer 2, last sentence).
-Reviewer 1 also raises an important technical point on the new data added in your revisions (points 2 and 3) that we agree you must include controls for figure 1C and 4A, and negative control region for ChIP-qPCR. Further you must address the point of this reviewer on comparison of regions between ChIP and RT-CPR (point 3).
-We also concur with Reviewer 1 that you must provide clarifications in the ms text for point 7 raised by the reviewer.
-Finally, we understand (from page 4 of your response letter) that your approach did not use exogenous spike-material, and therefore you cannot implement normalisation of the ChIPseq data. We recommend that you clarify in the next text and include this point from the reviewer as a potential limitation of your work.

Response:

We thank the editor and reviewers for their constructive comments. In this revision, we have substantially moderated the mechanistic interpretation of the study throughout the manuscript and revised the model figure to limit the conclusions to those directly supported by the evidence. We have also added and clarified controls for the ChIP-seq and ChIP-qPCR analyses. Specifically, we added an unsubtracted no-tag IP ChIP-seq heatmap using the same gene order as the Flag-tagged factor heatmaps, revised the RT-qPCR and Pol II ChIP-qPCR analyses to include overlapping loci and the FBP1 control locus, and added FBP1 as a low-signal control locus for the ChIP-qPCR analyses related to Fig. 4F/G. In addition, we clarified in the text that the ChIP-seq experiments were not performed with exogenous spike-in material and that these limits quantitative comparisons of absolute ChIP-seq signal between samples. We believe these revisions address the reviewers' technical concerns while more clearly defining the scope and limitations of the conclusions.

All changes in the revised manuscript are highlighted using tracked changes.

Reviewer #1 (Comments to the Authors (Required)):

In this revised version of the manuscript, the authors tried to respond to criticism from the reviewers by modifying the text and by including some additional results, focusing on the main points highlighted by the editor. However, the responses are limited and they do not reinforce the conclusions and do not allow to propose potential mechanisms.

Response:

We appreciate the reviewer's concern and agree that the revised data do not provide sufficient evidence to define a molecular mechanism. We have therefore reconsidered the overall framing of the manuscript. In the new revision, we no longer present the study as establishing a mechanism for Esc2/Rad57-dependent regulation of Pol II dynamics. Instead, we describe the findings more conservatively as evidence that Esc2 and Rad57 are associated with highly transcribed gene bodies and that *esc2*Δ *rad57*Δ cells show altered Pol II occupancy at a subset of these loci.

To address this point, we revised the Abstract, Introduction, Results, Discussion, and model figure. We removed or softened statements suggesting direct regulation of Pol II distribution, Pol II eviction/release, or a defined pathway that limits Pol II accumulation. We also added explicit statements that the biochemical state of the retained Pol II is not defined by the present data and that the mechanism remains unresolved. The model figure has been revised accordingly to summarize the observed associations rather than to propose a definitive mechanism.

Changes made in the revised manuscript

Because these revisions involved manuscript-wide changes in wording and interpretation, the corresponding changes are highlighted in the revised manuscript.

Specific points:

1. The major concerns remain unanswered on potential role of Esc2 and Rad57 in Pol II dynamics and transcriptional activity, and a potential link with growth phenotypes of deletion strains.

Response:

We thank the reviewer for raising this important point. We agree that the present data do not directly define the role of Esc2 and Rad57 in Pol II dynamics, transcriptional activity, or the growth phenotypes of the deletion strains. In particular, Pol II ChIP-seq and ChIP-qPCR measure chromatin occupancy but do not distinguish whether the increased Pol

II signal reflects altered elongation kinetics, stalled or slow-moving Pol II, non-productive retention, or another chromatin-associated state. We also agree that the slow-growth phenotype of *esc2*Δ *rad57*Δ cells is likely to be multifactorial and cannot be interpreted as a direct or exclusive consequence of altered Pol II occupancy.

To address this concern, we have substantially revised the manuscript to moderate the mechanistic interpretation. We no longer present Esc2 and Rad57 as direct regulators of Pol II dynamics or transcriptional activity. Instead, we describe the data more conservatively as showing that Esc2 and Rad57 are associated with highly transcribed gene bodies and that *esc2*Δ *rad57*Δ cells show altered/increased Pol II occupancy at a subset of these loci. We also revised the Discussion to state explicitly that the biochemical state of the retained Pol II is not defined by the present data and that the mechanism linking Esc2/Rad57 to altered Pol II occupancy remains unresolved.

We have also revised the text concerning growth phenotypes. In the revised manuscript, we state that the slow growth of *esc2*Δ *rad57*Δ cells likely reflects multiple contributing factors, including transcription-associated changes, mitochondrial DNA loss, and other physiological abnormalities, and that we do not interpret slow growth as a direct or exclusive consequence of altered Pol II occupancy.

Accordingly, we revised the Results and Discussion to avoid presenting the growth defect as a direct outcome of altered Pol II occupancy.

Changes made in the revised manuscript

Changes made in the revised manuscript: We revised the Abstract, the final paragraph of the Introduction, the Results sections describing Pol II occupancy and growth phenotypes, the Discussion, and the model figure/legend to moderate the mechanistic interpretation of Esc2/Rad57 function. These changes are highlighted in the revised manuscript.

Key changes include the revised Abstract (p. 2, lines 17–20), the revised Introduction framing (p. 5, lines 4–21), the revised interpretation of Pol II ChIP-seq/ChIP-qPCR data (pp. 9-10, lines 21–3), the revised discussion of the biochemical state of retained Pol II and the multifactorial nature of the growth phenotype (p. 16, lines 7–15, p. 17, lines 3–10), and the revised model figure and legend (Fig. 5B; p. 32, lines 1–12).

2. Even though the authors added no tag IP control in Fig 1A, B, this control is missing in other figures (for example Fig 1C, 4A etc) and is not included systematically in their analyses. Moreover, differences between ChIP signals for homologous-recombination components that the authors consider as enriched on highly transcribed genes and those that they consider as non-enriched are unclear. It is unclear how the authors conclude that Rrd1 is enriched, but

Rad51 and Rad55 or Ptc2 are non-enriched. A rigorous comparison with control IP condition is missing.

Response:

We thank the reviewer for pointing out that the no-tag IP control was not displayed systematically for the heatmap analyses. In the revised manuscript, we have added a new supplementary panel showing the unsubtracted no-tag IP ChIP-seq signal over the same gene set and gene order used for the Flag-tagged factor heatmaps in Fig. 1C, Fig. 4A, and Fig. S5C (new Fig. S1E). This no-tag IP dataset was used as the common background control for the ChIP-seq analyses of Flag-tagged Esc2, Rad57, Rad51, Rad55, Sit4, Rrd1, and Ptc2. We have also revised the figure legends and Methods to state explicitly that the Flag-tagged factor heatmaps were generated after subtraction of the common no-tag IP control.

We also agree that the distinction between factors showing gene-body-biased ChIP-seq profiles and those showing weaker or non-comparable profiles should be described more carefully. In the revised manuscript, we avoid presenting this as a simple binary classification. Instead, we describe Esc2, Rad57, and Sit4 as showing gene-body-biased profiles over highly transcribed genes that are distinguishable from the no-tag IP background. Rrd1 is now described more cautiously as showing a weaker and less sharply gene-body-centered profile than Esc2, Rad57, and Sit4. By contrast, Rad51, Rad55, and Ptc2 did not show comparable gene-body-biased profiles under the same analysis conditions. We have revised the Results text accordingly.

Changes made in the revised manuscript

We added a new no-tag IP ChIP-seq heatmap as Fig. S1E, generated over the same gene set and gene order used for the Flag-tagged factor heatmaps (p. 6, lines 21–23). We revised the corresponding figure legend to state that this no-tag IP dataset was used as the common background control for the ChIP-seq analyses of Esc2, Rad57, Rad51, Rad55, Sit4, Rrd1, and Ptc2.

We also revised the Results text, figure legends, and ChIP-seq Methods to state explicitly that the Flag-tagged factor heatmaps were generated after subtraction of the common no-tag IP control (p. 21, lines 9–15; Fig. 1C, Fig. 4A, Fig. S5C). In addition, we revised the Results text to avoid a simple enriched/non-enriched binary classification. In the genome-wide ChIP-seq analysis, Rrd1 is now described more cautiously as showing a weaker and less sharply gene-body-centered profile than Esc2, Rad57, and Sit4 (p. 11, lines 7–10). Rad51, Rad55, and Ptc2 are now described as not showing comparable gene-body-biased profiles under the same analysis conditions (p. 6, lines 11–23; p. 11, lines 10–12).

3. ChIP-qPCR and RT-qPCR results added on revision step (Fig 3F, G, 4F, G) were obtained for very few regions, only two regions that are not even the same between ChIP and RT-PCR on Fig 3F, G. The variability for Pol II ChIP-qPCR ratio on CDC19 (Fig 3G) is very high and incompatible with any possible conclusion and publication standard. No negative control region for ChIP-qPCR experiments was included that is needed to conclude on enrichment.

Response:

We thank the reviewer for pointing out these technical limitations. We agree that the previous ChIP-qPCR and RT-qPCR panels were based on too few loci, that the loci analyzed by ChIP-qPCR and RT-qPCR were not sufficiently matched, and that the variability of the *CDC19* Pol II ChIP-qPCR signal made this locus unsuitable for supporting the main conclusion. We have therefore substantially revised these panels. In the revised manuscript, we reconfigured Fig. 3F and Fig. 3G so that RT-qPCR and Pol II ChIP-qPCR include overlapping loci. Specifically, *RPL10, TPI1*, and *FBP1* are now analyzed in both assays, and *FBP1* was added as a control locus. *FBP1* was selected because our genome-wide Pol II ChIP-seq and RNA-seq datasets showed no increase in Pol II occupancy or RNA abundance at this locus in *esc2*Δ *rad57*Δ cells. *CDC19* has been removed from the main Pol II ChIP-qPCR panel and is no longer used to support the conclusion.

We also revised the presentation of the ChIP-qPCR data to show raw % input values with individual biological replicates and the mean, rather than relying on ratio-based or background-subtracted values. This presentation makes the signal intensity and variability transparent. In addition, for the ChIP-qPCR analyses related to Fig. 4F/G, we added *FBP1* as a low-signal control locus using the available ChIP DNA samples (new Fig. S5E). *FBP1* showed comparably low ChIP-qPCR signals across the available Flag-tagged strains and genetic backgrounds. Because only one Rrd1-4Flag WT ChIP sample was available for this control, we present these data as a supplementary low-signal control without statistical testing. We have revised the Results, figure legends, and Methods accordingly. The ChIP-qPCR Methods now state that signals are presented as raw % input values without subtraction of no-antibody or no-tag controls, and primer information for *FBP1* has been added to Table S6.

Changes made in the revised manuscript

We revised Fig. 3F and Fig. 3G so that RT-qPCR and Pol II ChIP-qPCR now include overlapping loci, with *RPL10, TPI1*, and *FBP1* analyzed in both assays. *FBP1* was added as a control locus, and *CDC19* was removed from the main Pol II ChIP-qPCR panel (p. 10, lines 4–9; Fig. 3F, G).

We revised the ChIP-qPCR presentation to show raw % input values with individual biological replicates and the mean, and updated the corresponding figure legends for Fig. 3G and Fig. 4F/G.

We also added *FBP1* as a low-signal control locus for the Fig. 4F/G ChIP-qPCR experiments, shown in new Fig. S5E (p. 12, lines 12–14; Fig. S5E). The ChIP-qPCR Methods were revised to state that signals are shown as raw % input values without subtraction of no-antibody or no-tag controls, and *FBP1* primer information was added to Table S6 (pp. 20-21, lines 22–2; Table S6).

4. *Since no appropriated normalization of ChIPseq data was done, different samples cannot be compared in a correct way to conclude about changes between strains. Moreover, Fig 3B for Pol II in double deletion mutant is problematic with very different baselines for three cluster metagene profiles.*

Response:

We thank the reviewer for raising this important point. We agree that, because the ChIP-seq experiments were not performed with exogenous spike-in material, the data do not allow rigorous quantitative comparison of absolute ChIP-seq signal levels between strains. We have therefore revised the manuscript to avoid overinterpreting global differences in absolute ChIP-seq signal intensity between WT and *esc2*Δ *rad57*Δ cells.

In the revised manuscript, we interpret the Pol II ChIP-seq data primarily in terms of relative occupancy patterns and gene-body distribution rather than absolute genome-wide changes in Pol II abundance. We also added an explicit limitation stating that the absence of spike-in normalization prevents definitive conclusions about absolute ChIP-seq signal differences between samples.

Regarding Fig. 3B, we agree that the different baselines among the metagene profiles complicate direct quantitative comparison between clusters. We have revised the corresponding Results text to avoid conclusions based on baseline differences and instead focus on the relative gene-body-biased pattern of Pol II signal in *esc2*Δ *rad57*Δ cells. We also support this interpretation with locus-specific Pol II ChIP-qPCR using raw % input values and the *FBP1* control locus in the revised Fig. 3G.

Changes made in the revised manuscript:

We revised the Results section describing Fig. 3B to state that the Pol II ChIP-seq datasets were not spike-in normalized and were therefore interpreted primarily in terms of relative Pol II distribution patterns rather than absolute differences in Pol II occupancy

between strains or clusters (p. 9, lines 12-14).

We also revised the cluster description to avoid conclusions based on baseline differences among the metagene profiles. Pol II-C1 and Pol II-C2 are now described as showing gene-body-biased Pol II signal increases, whereas Pol II-C3 is described as not showing a comparable gene-body-biased increase (p. 9, lines 14–20; Fig. 3B, C). We also added a limitation in the Discussion regarding the absence of spike-in normalization (p. 16, lines 7–10).

5. *The authors provided results on mitochondrial problems in esc2Δ rad57Δ that further reinforces the potential indirect effects unrelated to ChIPseq results.*

Response:

We thank the reviewer for this comment. We agree that the mitochondrial defects in *esc2*Δ *rad57*Δ cells may contribute indirectly to the transcriptome and growth phenotypes. We have therefore revised the manuscript to make this limitation explicit. In the revised Results and Discussion, we state that reduced mitochondrial transcripts are most consistent with secondary consequences of mitochondrial DNA loss, because Esc2 and Rad57 do not detectably associate with mitochondrial DNA, whereas *esc2*Δ *rad57*Δ cells lack mitochondrial nucleoids and fail to grow on glycerol medium.

We also revised the text to clarify that the slow-growth phenotype is likely multifactorial and is not interpreted as a direct or exclusive consequence of altered Pol II occupancy. The interpretation of the ChIP-seq and RNA-seq data has been moderated accordingly to avoid implying that all observed changes are direct effects of Esc2/Rad57 loss.

Changes made in the revised manuscript:

We revised the Results section describing mitochondrial phenotypes to state more explicitly that reduced mitochondrial transcripts in *esc2*Δ *rad57*Δ cells are most consistent with secondary consequences of mitochondrial DNA loss, rather than direct transcriptional regulation by Esc2/Rad57 at mitochondrial loci. This interpretation is based on the observations that Esc2 and Rad57 do not show detectable enrichment on mitochondrial DNA, whereas *esc2*Δ *rad57*Δ cells lack mitochondrial nucleoids and fail to grow on glycerol medium (p. 13, lines 3–11; Fig. S6B–D).

We also revised the Results section describing growth phenotypes to state that chronic loss of Esc2/Rad57 is associated with slow growth, but that this phenotype is likely multifactorial and is not interpreted as a direct or exclusive consequence of altered Pol II

occupancy (p. 13, lines 18–22).

In the Discussion, we added an explicit limitation stating that mitochondrial DNA loss, reduced mitochondrial transcripts, and slow growth may represent secondary or indirect consequences of combined Esc2/Rad57 deficiency. We further state that mitochondrial dysfunction and other genome-maintenance-related abnormalities may contribute to the physiological consequences of the double mutation, in addition to the transcription-associated changes described in the study (p. 17, lines 4–8).

6. The model figure contains many points without any evidence provided, for example for Pol II release by Rrd1 or reduced transcription (no direct measurements were done) or link with growth phenotypes.

Response:

We thank the reviewer for pointing out that several elements in the previous model figure were not directly demonstrated in the present study. We agree that our in vivo data do not directly measure Pol II release, reduced transcription, or a causal link between altered Pol II occupancy and growth phenotypes. Although previous work has implicated Rrd1 in the release of chromatin-associated Pol II, and our observations are consistent with the possibility that Sit4/Rrd1 may influence chromatin-associated Pol II, the present data do not establish direct Pol II eviction or release by Rrd1 in *esc2*Δ *rad57*Δ cells.

We have therefore revised the model figure to remove unsupported mechanistic steps, including direct Pol II release by Rrd1, reduced transcription, and a direct causal link to growth impairment. The revised model now summarizes the observations supported by our data: Esc2, Rad57, Sit4, and Rrd1 are associated with highly transcribed gene bodies, Sit4/Rrd1 association is reduced in *esc2*Δ *rad57*Δ cells at representative loci, and loss of Esc2/Rad57 is accompanied by altered Pol II occupancy. We also revised the Discussion to present a possible connection between Sit4/Rrd1 association and chromatin-associated Pol II, rather than Pol II release by Rrd1 as a conclusion.

Changes made in the revised manuscript:

We revised Fig. 5B and its legend to remove unsupported mechanistic elements, including direct Pol II eviction/release by Rrd1, reduced transcriptional output, and a causal link to growth impairment. The revised model summarizes only the observed associations among Esc2/Rad57/Sit4/Rrd1, highly transcribed gene bodies, reduced Sit4/Rrd1 association in *esc2*Δ *rad57*Δ cells, and altered Pol II occupancy (Fig. 5B; p. 32, lines 1–12).

We also revised the Discussion to state that the biochemical state of the retained Pol II remains unresolved and that our data do not directly demonstrate Pol II eviction or release by Rrd1. The relationship between Sit4/Rrd1 and chromatin-associated Pol II is now presented as a possible connection rather than as a demonstrated mechanism (pp. 17-18, lines 11–4).

7. *Supplementary figure panels are not always cited and the quality of some figures should be improved, for example Fig S2D. Replicate analysis reveals several problems, for example low correlation between WT r1 and r2 (0.55) even lower than between WT r1 and esc2 rad57 replicates (0.66, 0.62). The AA rad57 experience is difficult to interpret since there is a problem with -Rap replicate 2.*

Response:

We thank the reviewer for pointing out issues with supplementary figure citation, figure quality, and replicate consistency. In the revised manuscript, we checked the citation of all supplementary figure panels and added explicit citations where necessary. We also improved the resolution and presentation of the indicated supplementary panels.

We have also reorganized and clarified the supplementary QC panels to make the identity and purpose of each analysis clearer. The PCA and Pearson correlation analyses for the Esc2/Rad57/Rad51/Rad55 ChIP-seq datasets are now shown in Fig. S1C and Fig. S1D, whereas the RNA-seq PCA and correlation analyses are presented separately in Fig. S2A and Fig. S2B. The Pol II ChIP-seq PCA and correlation analyses are now explicitly cited in the Results as Fig. S3B and Fig. S3C.

We agree that the WT Pol II ChIP-seq replicates show greater variability. This may in part reflect the relatively low dynamic range and greater locus-to-locus fluctuation of gene-body Pol II signals in WT cells, although we cannot exclude technical variability. We therefore revised the manuscript to interpret these data cautiously and to avoid conclusions based on absolute signal differences between individual samples. Instead, we focus on reproducible gene-body-biased Pol II occupancy patterns in *esc2*Δ *rad57*Δ cells and support the locus-specific interpretation with Pol II ChIP-qPCR in the revised Fig. 3G.

Regarding the Rad57 anchor-away experiment, we agree that the RNA-seq dataset is difficult to interpret because one −Rap replicate showed markedly reduced correlation with the other samples. In the previous version, the differential expression analysis was performed using two −Rap and two +Rap samples. In the revised manuscript, we retain the full dataset for transparency, but we no longer use the anchor-away RNA-seq data to draw a strong

quantitative or mechanistic conclusion. We have revised the Results text and figure legend to state explicitly that this dataset should be interpreted cautiously because of replicate variability. Instead, we state more conservatively that acute Rad57 depletion in an *esc2*Δ background did not fully recapitulate the transcriptome changes observed in the stable *esc2*Δ *rad57*Δ mutant under the tested conditions.

Changes made in the revised manuscript:

We checked and corrected citations to all supplementary figure panels and improved the resolution and presentation of the indicated supplementary figures.

We reorganized the supplementary QC panels so that ChIP-seq and RNA-seq replicate analyses are clearly separated. Esc2/Rad57/Rad51/Rad55 ChIP-seq PCA and correlation analyses are now shown in Fig. S1C and Fig. S1D (p. 6, lines 16–21), RNA-seq PCA and correlation analyses are shown in Fig. S2A and Fig. S2B (pp. 7-8, lines 21–4), and Pol II ChIP-seq PCA and correlation analyses are explicitly cited in the Results as Fig. S3B and Fig. S3C (p. 9, lines 5–7).

We revised the Pol II ChIP-seq Results text to acknowledge the greater variability among WT replicates and to state that these data are interpreted cautiously, focusing on reproducible gene-body-biased occupancy patterns rather than absolute signal differences between individual samples (p. 9, lines 7–9; Fig. S3B, C).

We also revised both the Results text and the Fig. S7 legend for the Rad57 anchor-away RNA-seq experiment. The revised Results text states that the dataset should be interpreted cautiously because of replicate variability and that it is not used to draw a strong quantitative or mechanistic conclusion. The Fig. S7 legend now explicitly notes that one −Rap replicate showed markedly reduced correlation with the other samples and that the differential expression analysis should be interpreted cautiously (p. 14, lines 9–14, Fig. S7C–E legend, p. 37, lines 11–21).

Reviewer #2 (Comments to the Authors (Required)):

The authors have addressed the major points raised in my initial review. Specifically, they performed the requested genome-wide and gene-by-gene correlation analyses to directly test the relationship between Esc2/Rad57 occupancy and transcriptional output. As these new analyses revealed that the direct correlations are relatively weak or highly restricted, the

authors have revised the manuscript. However, the authors still imply a direct role for Esc2/Rad57 in regulating Pol II distribution during transcription (e.g., in the abstract). Because the data remains correlative, an active or mechanistic role for Esc2 and Rad57 in regulating Pol II dynamics is not fully supported. I recommend that the authors perform texts revision to soften these specific claims and their model.

Response:

We thank the reviewer for acknowledging that the major points raised in the initial review were addressed. We agree that our data remain correlative and do not establish a direct or active role for Esc2 and Rad57 in regulating Pol II dynamics during transcription. We have therefore revised the manuscript throughout to soften these claims and to align the interpretation more closely with the evidence.

In the revised Abstract, we no longer describe Esc2/Rad57 as directly regulating Pol II distribution or dynamics. Instead, we describe the findings in terms of associations between Esc2/Rad57, gene-body Pol II occupancy, and transcription-associated chromatin states.

We also revised the anchor-away statement to avoid concluding that acute Rad57 depletion causes only limited transcriptional changes; the revised text now states more cautiously that acute Rad57 depletion under the tested anchor-away conditions did not fully recapitulate the broader transcriptome imbalance and impaired growth observed after chronic Esc2/Rad57 loss. We also revised the final paragraph of the Introduction to avoid framing the study as demonstrating direct local control of Pol II distribution by Esc2/Rad57. The revised text now states that we examine the relationship between Esc2/Rad57 and Pol II occupancy and describes the findings as associations among recombination-related factors, gene-body Pol II occupancy, and transcription-associated chromatin states.

In the Results and Discussion, we replaced statements suggesting a direct regulatory pathway or active control of Pol II dynamics with more conservative wording. We now state that loss of Esc2/Rad57 is accompanied by altered Pol II occupancy, rather than concluding that these factors directly regulate Pol II distribution. We also explicitly state that the increased Pol II occupancy observed in *esc2*Δ *rad57*Δ cells does not define the biochemical state of the retained polymerase and does not distinguish among slow-moving, stalled, or otherwise non-productively retained Pol II.

Finally, we revised the model figure to remove unsupported mechanistic steps. In particular, the revised model no longer depicts direct Pol II eviction/release by Rrd1, reduced transcription, or a causal link to growth phenotypes. The model now summarizes the observations supported by our data: Esc2, Rad57, Sit4, and Rrd1 are associated with a gene-body chromatin context at highly transcribed genes, Sit4/Rrd1 association is reduced in

*esc2*Δ *rad57*Δ cells at representative loci, and loss of Esc2/Rad57 is accompanied by altered gene-body Pol II occupancy.

Changes made in the revised manuscript

Because these revisions involved manuscript-wide changes in wording and interpretation, the corresponding changes are highlighted in the revised manuscript.

September 17, 2026

RE: Life Science Alliance Manuscript #LSA-2026-03673-TRR

Dr. Hiroshi Masumoto
Nagasaki University
Biomedical Research Support Center (BRSC)
1-12-4 Sakamoto
Sakamoto
Nagasaki, Nagasaki 8528523
Japan

Dear Dr. Masumoto,

Thank you for submitting your revised manuscript entitled "Esc2 and Rad57 contribute to RNA polymerase II distribution at highly transcribed genes", and for your response to our questions regarding the technical concerns from Reviewer2 (appended below).

We have assessed the provided clarifications on methods and the modified Figure 3A (to swap TDH3 with a browser view for RPL10). Our opinion is that you have addressed the reviewer's concerns on normalisation before subtraction for the no-tag IP Control. With respect to the modification of Figure 3A, we note that it does not address the reviewer's point on TDH3 itself. We request that you retain TDH3 information and also include the browser view of of the gene, RPL10, as proposed in your correspondence

In light of the overall evaluation from Reviewer 2, we would be happy to publish your paper in Life Science Alliance pending (1) inclusion of new Figure 3A and associated changes to figure descriptions, (2) changes to reflect clarity in methods, and (3) final revisions necessary to meet our formatting guidelines. We suggest that you include your responses to the reviewer's comments in a separate document.

MANUSCRIPT ORGANIZATION AND FORMATTING:

To avoid unnecessary delays in the acceptance and publication of your paper, please read the following information carefully. Full guidelines are available on our Instructions for Authors page, https://www.life-science-alliance.org/authors

Submission of a paper that does not conform to Life Science Alliance guidelines will delay the acceptance of your manuscript.

-Please expand on imaging details to include information on objectives and filters for fluorescence imaging (line 488).
-Please add the X and Bluesky handles of your host institute/organization, as well as your own, and/or one of the authors, in our system.
-Please rename "Additional Information" to "Conflict of Interest."
-The contributions selected for Hironori Niki do not qualify them for authorship. Please either update the contributions in our system and in the Author Contributions section of the manuscript, or let us know if the authors need to be removed (and added eventually to the acknowledgment section).
-Please add callouts for Figure S3B, C, and Tables S1, S3-S5 to your main manuscript text.
-Please be sure that the authorship listing and order is correct.

We encourage our authors to provide original source data, particularly uncropped/-processed electrophoretic blots and spreadsheets for the main figures of the manuscript. If you would like to add source data, we would welcome one PDF/Excel-file per figure for this information. These files will be linked as supplementary "Source Data" files.

It is Life Science Alliance policy that if requested, original data images must be made available to the editors. Failure to provide original images upon request will result in unavoidable delays in publication. Please ensure that you have access to all original data images prior to final submission.

If you are planning a press release on your work, please inform us immediately to allow informing our production team and scheduling a release date.

We welcome submissions of potential cover images for the issue of LSA in which your work would appear. If you have high quality images associated with this work, please feel free to email these, with a caption, to the journal office.

LSA encourages authors to provide a 30-60 second video where the study is briefly explained. These videos will be appear embedded with the manuscript online at Life Science Alliance, and on social media to promote the published paper and authors (for examples, see https://docs.google.com/document/d/1-UWCfbE4pGcDdcgzcmiuJl2XMBJnxKYeqRvLLrLSo8s/edit?usp=sharing). Corresponding or first-authors are welcome to submit the video. Please submit only one video per manuscript. The video can be emailed to contact@life-science-alliance.org

FINAL FILES:

To upload the final version of your manuscript, please log in to your account: https://lsa.msubmit.net/cgi-bin/main.plex
You will be guided to complete the submission of your revised manuscript and to fill in all necessary information. Please get in touch in case you do not know or remember your login name.

The following items are required for acceptance.

-- An editable version of the final text (.DOC or .DOCX) is needed for copyediting (no PDFs).

-- High-resolution figure, supplementary figure and video files uploaded as individual files: See our detailed guidelines for preparing your production-ready images, https://www.life-science-alliance.org/authors

-- Summary blurb (enter in submission system): A short text summarizing in a single sentence the study (max. 200 characters including spaces). This text is used in conjunction with the titles of papers, hence should be informative and complementary to the title. It should describe the context and significance of the findings for a general readership; it should be written in the present tense and refer to the work in the third person. Author names should not be mentioned.

The license to publish form must be signed before your manuscript can be sent to production. A link to the license to publish form will be available to the corresponding author only. Please take a moment to check your funder requirements.

Reviews, decision letters, and point-by-point responses associated with peer-review at Life Science Alliance will be published, alongside the manuscript. If you do want to opt out of having the reviewer reports and your point-by-point responses displayed, please let us know immediately.

Thank you for your attention to these final processing requirements. Please revise and format the manuscript and upload materials as soon as you are able.

Thank you for this interesting contribution to the literature. We look forward to publishing your paper in Life Science Alliance.

Sincerely,

Sarita Hebbar, PhD
Scientific Editor
Life Science Alliance
http://www.lsajournal.org

Reviewer #2 (Comments to the Authors (Required)):

The authors have moderated the claims and adjusted the models as requested. I recommend this manuscript for publication.

qRT-PCR: The authors have included the RPL10 and TPI1 genes in the Pol II ChIP-qPCR analysis (and FBP1 as a negative control), but only RPL10 showed a clear increase in Pol II occupancy in the double mutant. This is somewhat surprising, since the ChIP-seq data should have allowed the authors to identify genes with increased Pol II occupancy in the double mutant, or at least to confirm the gene for which they show the read distribution in Fig. 3A (TDH3).

no tag IP control and subsequent subtraction: A no-tag IP background subtraction was applied to the ChIP-seq analysis, which is an important and appreciated control. However, I note that the new Suppl. Fig. 1E shows that the no-tag background is heavily enriched at genes very similar to those enriched for Esc2 and Rad57. If the same number of reads were obtained for the no-tag IP and the Flag-tagged datasets, I believe this should not be an issue. However, if the no-tag IP and Flag-tagged datasets had different sequencing depths and no normalization was applied prior to subtraction, this could potentially introduce a bias.

Rebuttal
September 24, 2026

Dear Prof. Sarita Hebbar and Reviewers,

Thank you for forwarding the reviewer's additional comments. We are pleased that the reviewer recommends the manuscript for publication, and we appreciate the opportunity to clarify the remaining technical points.

We have carefully reviewed the reviewer's concerns and have made the following additional revisions.

First, regarding Fig. 3A and the Pol II ChIP-qPCR validation, we have revised Fig. 3A to retain the *TDH3* browser view and to additionally include the *RPL10* browser view, as requested. *RPL10* shows increased Pol II occupancy in the ChIP-seq browser view and is also the locus that shows a clear increase by Pol II ChIP-qPCR in Fig. 3G. This revision therefore aligns the representative ChIP-seq locus with the qPCR validation while retaining the *TDH3* information from the original figure. We also note that *TPI1* shows little or no increase by ChIP-qPCR, consistent with our revised interpretation that altered Pol II occupancy in *esc2*Δ *rad57*Δ cells is locus-dependent rather than uniform across all highly transcribed genes.

Second, regarding the no-tag IP control and subtraction, we would like to clarify that subtraction was not performed using raw sequencing read counts. All no-tag IP and Flag-tagged ChIP-seq datasets were first converted to bigWig files using CPM normalization with deepTools bamCoverage. The no-tag IP subtraction was then performed using these CPM-normalized bigWig files. Thus, differences in sequencing depth between no-tag IP and Flag-tagged datasets were accounted for before subtraction. Similarly, the Pol II ChIP-seq browser tracks shown in Fig. 3A were generated from CPM-normalized bigWig files after subtraction of the no-antibody IP control.

We also rechecked the sequencing-depth information for the common no-tag IP datasets using the FastQC reports and found a typographical error in Table S1. The total read number for noIP_2 had inadvertently been duplicated from noIP_1. The correct total read numbers are 12,854,887 for noIP_1 and 14,910,255 for noIP_2. We have corrected Table S1 accordingly. This correction does not affect the ChIP-seq profiles or the no-tag IP subtraction, because the analyses were performed using the original FASTQ files and CPM-normalized bigWig files, not the values listed in Table S1.

We have also revised Table S1 to include only the ChIP-seq datasets relevant to the analyses shown in

the revised manuscript. Unrelated Pol II CTD S2P/S5P datasets were removed to avoid confusion.

In addition, we revised the Methods to state explicitly that coverage tracks were generated using CPM normalization and that no-tag IP or no-antibody IP subtraction was performed using CPM-normalized bigWig files, not raw read counts.

We also incorporated the requested final formatting and reporting changes, including the additional fluorescence-imaging details, revised author-contribution information, and the requested supplementary figure and table callouts.

September 29, 2026

RE: Life Science Alliance Manuscript #LSA-2026-03673-TRRR

Dr. Hiroshi Masumoto
Nagasaki University
Biomedical Research Support Center (BRSC)
1-12-4 Sakamoto
Sakamoto
Nagasaki, Nagasaki 8528523
Japan

Dear Dr. Masumoto,

Thank you for submitting your Research Article entitled "Esc2 and Rad57 contribute to RNA polymerase II distribution at highly transcribed genes". It is a pleasure to let you know that your manuscript is now accepted for publication in Life Science Alliance. Congratulations on this interesting work.

The final published version of your manuscript will be deposited by us to PubMed Central upon publication.

Your manuscript will now progress through copyediting and proofing. It is journal policy that authors provide original data upon request.

Your article will publish open access upon publication under a CC-BY license.

Reviews, decision letters, and point-by-point responses associated with peer-review at Life Science Alliance will be published, alongside the manuscript. If you do want to opt out of having the reviewer reports and your point-by-point responses displayed, please let us know immediately.

IMPORTANT: If you will be unreachable at any time, please provide us with the email address of an alternate author. Failure to respond to routine queries may lead to unavoidable delays in publication.

Scheduling details will be available from our production department. You will receive proofs shortly before the publication date. Only essential corrections can be made at the proof stage so if there are any minor final changes you wish to make to the manuscript, please let the journal office know now.

DISTRIBUTION OF MATERIALS:
Authors are required to distribute freely any materials used in experiments published in Life Science Alliance. Authors are encouraged to deposit materials used in their studies to the appropriate repositories for distribution to researchers.

You can contact the journal office with any questions, contact@life-science-alliance.org

Again, congratulations on a very nice paper. I hope you found the review process to be constructive and are pleased with how the manuscript was handled editorially. We look forward to future exciting submissions from your lab.

Sincerely,

Sarita Hebbar, PhD
Scientific Editor
Life Science Alliance
http://www.lsajournal.org
